# Cycloruthenated Self‐Assembly with Metabolic Inhibition to Efficiently Overcome Multidrug Resistance in Cancers

**DOI:** 10.1002/adma.202100245

**Published:** 2021-10-24

**Authors:** Jia Li, Leli Zeng, Zheng Wang, Hengxing Chen, Shuo Fang, Jinquan Wang, Chao‐Yun Cai, Enming Xing, Xinxing Liao, Zhi‐Wei Li, Charles R. Ashby, Zhe‐Sheng Chen, Hui Chao, Yihang Pan

**Affiliations:** ^1^ Guangdong Provincial Key Laboratory of Digestive Cancer Research Precision Medicine Center The Seventh Affiliated Hospital Sun Yat‐Sen University Shenzhen Guangdong 518107 P. R. China; ^2^ MOE Key Laboratory of Bioinorganic and Synthetic Chemistry School of Chemistry Sun Yat‐Sen University Guangzhou 510275 P. R. China; ^3^ College of Pharmacy and Health Sciences St. John's University New York NY 11439 USA; ^4^ College of Chemistry and Chemical Engineering Key Laboratory of Chemical Additives for China National Light Industry Shaanxi University of Science and Technology Xi'an 710021 P. R. China; ^5^ Guangdong Province Key Laboratory for Biotechnology Drug Candidates School of Bioscience and Biopharmaceutics Guangdong Pharmaceutical University Guangzhou 510006 P. R. China

**Keywords:** antitumor agents, cyclometalated ruthenium complexes, metabolism inhibition, multidrug resistance, self‐assembly

## Abstract

The synthesis and the evaluation of the efficacy of a cycloruthenated complex, RuZ, is reported, to overcome multi‐drug resistance (MDR) in cancer cells. RuZ can self‐assemble into nanoaggregates in the cell culture medium, resulting in a high intracellular concentration of RuZ in MDR cancer cells. The self‐assembly significantly decreases oxygen consumption and inhibits glycolysis, which decreases cellular adenosine triphosphate (ATP) levels. The decrease in ATP levels and its low affinity for the ABCB1 and ABCG2 transporters (which mediate MDR) significantly increase the retention of RuZ by MDR cancer cells. Furthermore, RuZ increases cellular oxidative stress, inducing DNA damage, and, in combination with the aforementioned effects of RuZ, increases the apoptosis of cancer cells. Proteomic profiling analysis suggests that the RuZ primarily decreases the expression of proteins that mediate glycolysis and aerobic mitochondrial respiration and increases the expression of proteins involved in apoptosis. RuZ inhibits the proliferation of 35 cancer cell lines, of which 7 cell lines are resistant to clinical drugs. It is also active in doxorubicin‐resistant MDA‐MB‐231/Adr mouse tumor xenografts. To the best of our knowledge, the results are the first to show that self‐assembled cycloruthenated complexes are efficacious in inhibiting the growth of MDR cancer cells.

## Graphical abstract

The cyclometalated Ru(II) complex, RuZ, is reported to be able to self‐assemble and accumulate in high levels in multi‐drug resistant cancer cells. RuZ significantly increases cancer cell death by dual metabolic inhibition and interactions with multiple biological targets. Overall, the results suggest that RuZ could represent a potential treatment for cancers including multi‐drug resistant cancers.

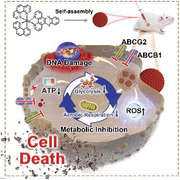

## 1 Introduction

Currently, one of the major impediments for the eradication of cancerous tumors is multi‐drug resistance (MDR).^[^ ^1^ ^]^ It has been shown that cancer cells can become MDR due to a number of mechanisms. One of the primary mediators of MDR in cancer cells is the overexpression of specific efflux transporters, which attenuate or even abrogate the efficacy of various anticancer drugs.^[^ ^2^ ^]^ Numerous studies have shown that the overexpression of the adenosine triphosphate (ATP) binding cassette (ABC) transporters, ABCB1 (i.e., MDR1/P‐glycoprotein‐P‐gp) or ABCG2 (i.e., Breast cancer resistance protein‐BCRP/Mitoxantrone resistance‐MXR), produces MDR in various types of cancers.^[^ ^3^ ^]^ Indeed, cancer cells can also develop MDR by evading various forms of cell death, such as apoptosis, necroptosis and ferroptosis.^[^ ^4^ ^]^ Importantly, the evasion of cell death and the ABC transporter‐mediated efflux of anticancer drugs have been reported to be energy‐dependent.^[^ ^5^ ^]^ Mechanistic studies suggest that drug‐resistant cancer cells have an increase in the activity of certain metabolic pathways that provides the energy required to mediate viability and drug resistance.^[^ ^6^ ^]^ For example, increased levels of ATP increase the efflux of anticancer drugs by ABC transporters,^[^ ^7^ ^]^ and an increase in glycolysis decreases apoptosis.^[^ ^8^ ^]^ Therefore, the development of drugs that target cancer cell metabolism may represent a promising approach to overcome MDR.

It has been reported that certain metal complexes can inhibit certain metabolic pathways in some parental (i.e., drug‐sensitive) cancer cell lines. For example, Mao et al. reported that iridium(III) complexes can inhibit mitochondrial oxidative respiration in A549 cancer cells.^[^ ^9^ ^]^ Furthermore, Guo et al. reported the synthesis of a platinum(II) complex that inhibited aerobic mitochondrial respiration and cytoplasmic glycolysis in Caov3 cancer cells.^[^ ^10^ ^]^ The majority of metal complexes are lipophilic cations that can readily cross the lipophilic cell membrane to target mitochondria and other organelles.^[^ ^11^ ^]^ However, MDR cancer cells have been shown to have a distinct membrane composition compared to the parental cancer cells. The ABCB1 and ABCG2 transporters are overexpressed in certain MDR cancer cells compared to their corresponding parental cells.^[^ ^12^ ^]^ Furthermore, certain metal complexes can be extruded from cancer cells due to the overexpression of some transporters.^[^ ^13^ ^]^ Thus, designing metal complexes 1) that disrupt cancer cell metabolism and 2) escape drug efflux by ABC transporters could yield molecules that are efficacious in MDR cancer cells.

Recently, assembly‐driven molecular aggregation has been used for synthesizing nanoparticles.^[^ ^14^ ^]^ Small molecular drugs, primarily via non‐covalent interactions, can spontaneously selfassemble into nanodrugs in aqueous solutions.^[^ ^15^ ^]^ Several cyclometalated complexes have been reported to selfassemble into nanoparticles and produce anticancer efficacy. Che and Bonnet reported that the cyclometalated platinum/gold/palladium complex forms supramolecular self‐assembly complexes via intermolecular interactions, producing an increase in drug efficacy.^[^ ^16^ ^]^ Thus, it is possible that the nanoscale self‐assembly of cyclometalated compounds could deliver high concentrations of a drug to the drug‐resistant cancer cells by providing a high drug‐loading capacity.

**Scheme 1:**
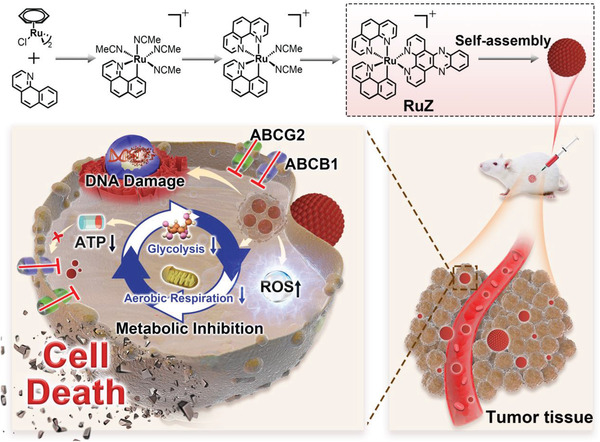
Chemical structures of the ligands and the synthetic scheme for RuZ, and the design of RuZ.

In contrast to the above‐mentioned cyclometalated platinum/gold/palladium compounds with tetra‐coordinated structures (Figure S1, Supporting Information), cyclometalated Ru(II) complexes have a significant octahedral character due to hexa‐coordination,^[^ ^17^ ^]^ thereby making Ru(II) complex a large multidimensional structure that interacts with multiple biological targets.^[^ ^18^ ^]^ Although single cyclometalated Ru(II) compounds have been reported to have anticancer efficacy,^[^ ^19^ ^]^ there have been no reports of self‐assembled cycloruthenated complexes. In this study, we designed a cyclometalated Ru(II) complex, RuZ, to overcome MDR in cancer cells, using self‐assembly (**Scheme** **1**). RuZ was synthesized using a three‐step synthesis method by coordinating three common, flat aromatic ligands that essentially have no extra functional group. RuZ can spontaneously self‐assemble into nano‐scale aggregates in water that are stable in the cell culture medium. This strategy for overcoming MDR in cancer cells has several advantages: 1) the three large aromatic ligands have a strong π–π stacking interactions for self‐assembly with a high drug‐loading rate; 2) a typical octahedral geometry with no extra functional groups, which decreases its interaction with the ABCB1 and ABCG2 transporters, thereby increasing its retention in drug‐resistant cancer cells; 3) a deprotonated C,N‐cyclometalated ligand, benzo[h]quinolone (bzq), that has an increased electron density at the metal center, producing redox chemistry activity and 4) a lipophilic cation structure with a dppz ligand that can be inserted into double‐stranded DNA molecules, which contributes to targeting mitochondria and nuclei, altering cancer metabolism.

## 2 Results and Discussion

**Figure 1:**
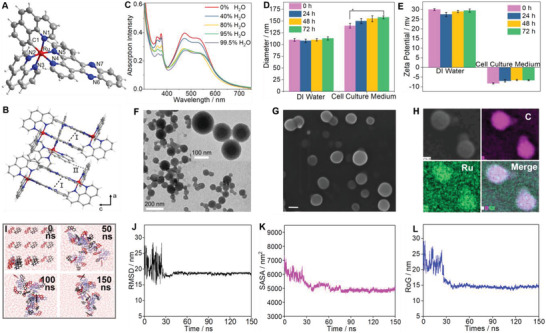
A) The X‐ray crystal structure of RuZ with 40% thermal ellipsoids (C, gray; N, blue; Ru, red), PF_6_^−^ has been omitted for clarity. B) Interactions between adjacent molecules of RuZ. C) Absorption spectra of R uZ (10 × 10^−6^ m) in DMSO and after the addition of water (0% to 99.5%). D) The mean hydrodynamic diameter of RuZ in deionized (DI) water or cell culture medium (DMEM + 10% FBS), *p < 0.05. E) The mean zeta potential of RuZ in DI water or cell culture medium. F) TEM scan of 100 × 10^−6^ m of RuZ in DI water. Scale bar: 200 nm. G) SEM scan of 100 × 10^−6^ m of RuZ in DI water. Scale bar: 100 nm. H) SEM elemental mapping of 100 × 10^−6^ m RuZ in DI water. I) MD simulation snapshots for RuZ system in aqueous phase, 0, 50, 100, and 150 ns. J) RMSD, K) SASA, and L) mass‐weighted radius of gyration versus time analysis results for RuZ.

As shown in Scheme S1, RuZ was synthesized according to a previously published method.^[^ ^19e^ ^]^ RuZ was obtained as a dark‐purple solid and was purified using column chromatography (yield: 40%). RuZ was characterized using ESI‐MS, HRMS, HPLC, ^1^H NMR, ^13^C NMR, elemental analysis and single‐crystal X‐ray diffraction (Figures S2–S6, Supporting Information). RuZ had a monocationic ESI spectrum and RuZ was not phosphorescent at room temperature due to the cyclometallation of bzq (Figure S7, Supporting Information), with the stabilized, triplet excited state conforming to the energy gap law. Furthermore, the σ donating capacity of the carbon anion in the bzq ligand increases the ligand field, giving RuZ a broad absorption spectra in the range of 400–600 nm. Single crystals of RuZ were obtained by vapor diffusion going from diethyl ether to a MeCN/CH_2_Cl_2_ solution in the presence of KPF_6_. The crystallographic data are shown in Tables S3 and S4 (Supporting Information). As shown in **Figure** **1**A, five nitrogen atoms and one carbon atom are coordinated to ruthenium in the reference complex, RuZ (PF_6_) and the ruthenium ion of RuZ is in a slightly distorted octahedral environment. As expected, there was an elongation of the Ru—N bond, as compared to the Ru—C bond (Tables S4, Supporting Information). In the packing structure (Figure 1B and Figure S8: Supporting Information), adjacent RuZ molecules are primarily connected by π–π interactions (I) between the dppz ligands and C–H π interactions (II).

In a 2D diffusion‐ordered ^1^H NMR spectroscopy (DOSY) experiment, the presence of the same band of RuZ, at a high concentration, compared to low concentration, confirmed that a single pure product was formed (Figure S9, Supporting Information). The measured weight‐average diffusion coefficients were 4.25 × 10^−4^ m^2^ s^−1^ for the low concentration of RuZ and 3.99 × 10^−4^ m^2^ s^−1^ for the high concentration RuZ, which suggests a large molecular size at a high concentration and the formation of self‐assembly under solution conditions.^[^ ^20^ ^]^ We also investigated the self‐assembly of RuZ in water. The absorption of RuZ in H_2_O/DMSO mixtures are shown in Figure 1C. When the H_2_O content is increased to 99.5%, the absorption peak decreased. RuZ produced Mie light scattering that may be due to the molecular aggregates. As shown in Figure S10 (Supporting Information), there is an obvious Tyndall effect under laser irradiation following the addition of a DMSO stock solution into a water solution (99.5% H_2_O, v/v), suggesting the generation of aggregates. In contrast, there was no Tyndall effect in the pure DMSO solution. The RuZ self‐assembly in the water medium was also characterized using dynamic light‐scattering (DLS). The resulting histogram indicated an average hydrodynamic radius of the aggregates of ≈110 nm in deionized (DI) water (Figure 1D) and the aggregates had an average of radius of ≈135 nm in the cell culture medium. Moreover, the average hydrodynamic radius of the RuZ nanoparticles in PBS was > 300 nm, which was greater than that of RuZ nanoparticles in DI water (Figure S11, Supporting Information). We hypothesize that the phosphate ion could influence the aggregation of RuZ nanoparticles. There was a slight increase in the hydrodynamic radius after 72 h of incubation, suggesting that RuZ nanoparticles are not stable in PBS, compared to DI water and the cell culture medium. The zeta potential of the self‐assembly solution was decreased from ≈30 to −8.1 mV when the DI water solution was replaced with the cell culture medium (Figure 1E). TEM and SEM experiments also indicated the self‐assembly of RuZ (Figure 1F,G). The TEM and SEM images indicated that the RuZ nanoparticles in DI water had good monodispersity and a similar diameter to RuZ self‐assembly in DLS. The self‐assembly formation was further confirmed by SEM elemental mapping (Figure 1H), as indicated by the distribution of Ru and C elements in the same particle. Furthermore, the self‐assembly process of RuZ in water was investigated using Discovery Studio 2016 Client software.^[^ ^21^ ^]^ As shown in Figure 1I, twelve RuZ molecules were placed in a water box. All 12 molecules aggregated after 150 ns simulations due to intermolecular interactions. Based on the curve of root‐mean‐squared deviation (RMSD), the system remained stable after ≈40 ns (Figure 1J). The solvent accessible surface areas (SASA) and radius of gyration (RoG) of the system decreased after the first 40 ns, confirming the self‐assembly trend of the system (Figure 1K,L). Overall, these results indicated that a single RuZ complex can spontaneously self‐assemble into nanoaggregates in water medium.

The cytotoxicity of RuZ was determined in 35 cancer cell lines (Figure S12: Supporting Information and Table S1). For comparison, the cytotoxicity of cisplatin was also determined in the same cancer cell lines. Overall, the IC_50_ values of RuZ in the parental cancer cell lines ranged from 0.25 to 4.0 × 10^−6^ m (Figure S12, Supporting Information) and RuZ was 2‐ to 60‐fold more potent than cisplatin. RuZ was cytotoxic in a wide range of cancer cell lines, and was most efficacious in H23, H460, SW620, COLO‐205, SF‐539, SK‐MEL‐28, T‐47D, MDA‐MB‐231 and HeLa cancer cell lines, with IC_50_ values from 0.25–1.0 × 10^−6^ m. RuZ was less efficacious in HCT‐15 and CAKI‐1 compared to the other cancer cell lines. RuZ was significantly less cytotoxic in the normal cell lines, LO2 (human liver) and MCF10A (mammary epithelial cells), compared to the cancer cell lines (Table S1, Supporting Information), indicating that RuZ is relatively selective for the cancer cells and may have a lower probability of producing toxicity in normal cells.

The efficacy of RuZ in seven parental cancer cell lines and their corresponding chemoresistant sublines was also determined (**Table** **1**). For comparison, the clinically approved anticancer drugs, doxorubicin (Dox), mitoxantrone (MX), cisplatin (Pt) and arsenic trioxide (As_2_O_3_), were used to determine drug resistance (Table S2, Supporting Information). In the MX‐resistant H460/MX20 cells,^[^ ^22^ ^]^ that had a resistance‐fold (RF) of 55‐fold for MX, the efficacy of RuZ was similar to the corresponding parental H460 cancer cells. The efficacy of cisplatin was decreased by 10‐fold and 8.5‐fold in the cisplatin‐resistant cancer cell lines, BEL‐7404/CP20 and BIU‐87/DDP, respectively, compared to the parental BEL‐7404 and BIU‐87 cancer cell lines. In contrast, RuZ was efficacious both in the drug‐resistant and parental cancer cells, with RF values of 0.98‐ and 1.58‐fold, respectively, in BEL‐7404 and BIU‐87 drug‐resistant and parental cancer cell lines. The results suggest that RuZ was highly efficacious in the cisplatin‐resistant cancer cell lines used in this study. RuZ was equally efficacious in the As_2_O_3_‐resistant cancer cells,^[^ ^23^ ^]^ KB/ATO, and the parental KB‐3‐1 cells. To the best of our knowledge, RuZ is the first ruthenium complex known to have in vitro efficacy in As_2_O_3_‐resistant cancer cells. The RF of RuZ in the ABCB1‐overexpressing cell lines, KB‐C2 and SW620/AD300, was only 1.5 and 1.8, respectively, compared to an RF of 30‐35‐fold, respectively, for Dox, an ABCB1 substrate. In the Dox‐resistant MDA‐MB‐231/Adr cancer cells, which had an RF of 37‐fold for Dox, RuZ was also efficacious, with an IC_50_ value of 1.96 × 10^−6^ m. Overall, our results indicate that RuZ is efficacious in several cancer cells that confer resistance to certain clinically used anticancer drugs.

**Table 1:** The efficacy of RuZ after 48 h of incubation in six types of parental cancer cell lines and their drug‐resistant sublines

Cell Lines	RuZ^d)^ [× 10^−6^ m]	Resistance Fold (RF)^a)^	Resistance Mechanisms
H460	0.67 ± 0.12		Parental
H460/MX20	0.70 ± 0.27	1.04	ABCG2
SW620	0.97 ± 0.11		Parental
SW620/AD300	1.75 ± 0.63	1.80	ABCB1
KB‐3‐1	1.76 ± 0.16		Parental
KB‐C‐2	2.64 ± 0.57	1.50	ABCB1
KB/ATO	1.58 ± 0.10	0.90	*N* ^b)^
BEL‐7404	1.36 ± 0.22		Parental
BEL‐7404/CP20 BIU‐87 BIU‐87/DDP	1.34 ± 0.46 0.85 ± 0.45 1.26 ± 0.57	0.98 1.58	*M* ^c)^ Parental *M*
MDA‐MB‐231	1.25±0.31		Parental
MDA‐MB‐231/Adr	1.96±0.24	1.57	MDR

^a)^ RF was calculated by dividing the IC_50_ value for the resistant cell by the IC_50_ value for parental cell

^b)^ *N* indicates no clear mechanisms

^c)^ *M* indicates complicated mechanisms

^d)^ The values for RuZ represent the mean concentration of RuZ required to inhibit cell viability by 50% (IC_50_) (*n* = 3, mean ± SD).

**Figure 2:**
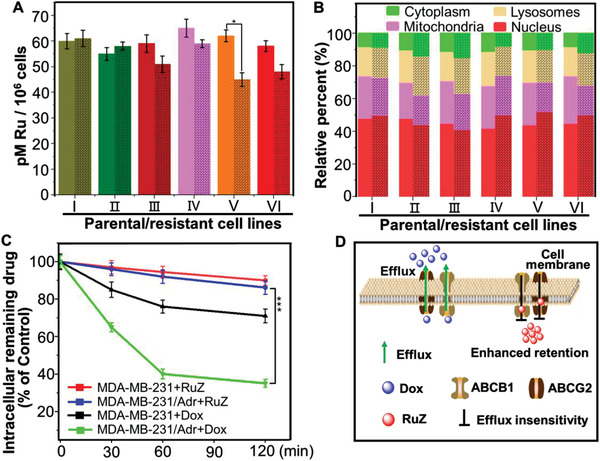
A) The uptake of RuZ in six groups of parental cancer cell lines and their drug‐resistant cell lines (black grid), I: KB‐3‐1 and KB/ATO; II: BEL‐7404 and BEL‐7404/CP20; III: BIU‐87 and BIU‐87/DDP; IV: H460 and H460/MX20; V: SW620 and SW620/AD300; VI: MDA‐MB‐231 and MDA‐MB‐231/Adr. B)The subcellular distribution of ruthenium in parental and drug‐resistant cancer cells after incubation with RuZ (2.5 × 10^−6^ m) for 2 h. C) The efflux of drugs in MDA‐MB‐231 and MDA‐MB‐231/Adr cells after incubation with Dox (1.0 × 10^−6^ m) or RuZ (2.5 × 10^−6^ m). ***p < 0.0005. D) A schematic showing the efflux of Dox and RuZ by the efflux transporters ABCG2 and ABCB1.

The efficacy of anticancer drugs is dependent on their intracellular drug accumulation, especially in MDR cancer cells. Therefore, we compared the uptake of RuZ to specific anticancer drugs in the aforementioned six groups of parental and drug‐resistant cancer cells. As shown in **Figure** **2**A, there was no significant difference in the intracellular accumulation of ruthenium between the parental and drug‐resistant cancer cells in group I (KB‐3‐1 and KB/ATO cancer cells), and group IV (H460 and H460/MX20 cancer cells). However, the intracellular levels of MX and arsenic (As) in the drug‐resistant cells were 5‐to 9‐fold lower than that of the parental H460 and KB‐3‐1 cells (Figure S13A, Supporting Information), suggesting a dereased uptake of MX and arsenic in the drug‐resistant cancer cells. For the cisplatin‐resistant cancer cells in group II and III, there was a 6‐fold decrease in platinum uptake in BEL‐7404/CP20 and BIU‐87/DDP compared to their respective parental cancer cell lines. However, there was no significant difference in the uptake of ruthenium between the cisplatin‐resistant and the parental cancer cell lines. However, the BEL‐7404/CP20 cancer cells accumulated a significantly greater amount of ruthenium compared to BIU‐87/DDP cancer cells and thus, we hypothesize that RuZ may be more efficacious in inhibiting the growth of BIU‐87/DDP cancer cells. The intracellular level of Dox in the MDR cancer cell lines (group V and VI), SW620/AD300 and MDA‐MB‐231/Adr, was 15‐fold less than that of their parental SW620 and MDA‐MB‐231 cells, respectively, indicating a high magnitude of drug resistance. In contrast, the levels of ruthenium in the two resistant cancer cell lines were less than 1.5‐fold compared to the parental cancer cell lines, suggesting that RuZ is also efficacious in specific cancer cells resistant to Dox. Furthermore, as predicted, there was a significant reduction in the accumulation of cisplatin, MX and Dox in drug‐resistant cancer cells. Although the intracellular levels of ruthenium were relatively low in SW620/AD300 and MDA‐MB‐231/Adr cells, RuZ was significantly more efficacious in the drug‐resistant cancer cells compared to the cancer cells incubated with cisplatin, As_2_O_3_, MX or Dox.

Based on the above results, we determined the distribution of ruthenium in cellular organelles. As shown in Figure 2B, there was no significant difference in the subcellular distribution of ruthenium in the six groups of drug‐resistant and parental cancer cell lines, where the % accumulation of ruthenium alone was 40–50% in the nuclei, 30% in the mitochondria, 15% in the lysosomes and 10% in the cytoplasm. There was a small, non‐significant decrease in the accumulation of ruthenium in the mitochondria and a non‐significant increase in the nuclei of the drug‐resistant cancer cells compared to the parental cells. These results suggest that the subcellular distribution of RuZ is mainly determined by the structure of the Ru(II) complex. Furthermore, RuZ is a lipophilic molecule, with a positive charge and log*P*_o/w_ value of 1.7, which helps it to target mitochondria.^[^ ^24^ ^]^ Importantly, the inserting ligand, dppz, facilitated the insertion of RuZ into double‐stranded DNA, thereby increasing its nuclei targeting efficacy.

To determine the cellular uptake mechanism of RuZ, the amount of ruthenium in MDA‐MB‐231/Adr cancer cells was measured following incubation with RuZ for 2 h under normal conditions, 4°C or preincubated with 2‐deoxy‐d‐glucose (a metabolic inhibitor) and chloroquine and NH_4_Cl (endocytosis inhibitors), using ICP‐MS analysis. As shown in Figure S13B (Supporting Information), significant variations in the ruthenium uptake occurred after preincubation with the inhibitors and at 4°C compared to the control group (RuZ), suggesting that an energy‐dependent pathway was mediating the uptake of RuZ. As nanomaterials are typically taken up by endocytosis, our results further indicated that RuZ forms nanoaggregates that can be transported into cells by endocytosis.

The Molecular Autodock program was used to obtain additional information about the interactions between RuZ and the efflux transporters, ABCG2 and ABCB1, by calculating docking scores for RuZ at the transmembrane domain (TMD) of the ABCG2 and ABCB1 transporters.^[^ ^25^ ^]^ Higher absolute values of the binding score indicate a more favorable interaction with the target. For comparison, Dox, a known substrate of the ABCG2 and ABCB1 transporters, was used as a positive control drug. As shown in Figure S14 (Supporting Information), Dox had a docking score of −9.37 and −9.01 kcal mol^−1^ in the TMD of the ABCG2 and ABCB1 transporters, respectively. These relatively high absolute values indicated a significant interaction between Dox and the ABCG2 and ABCB1 transporters. It is well known that Dox is a substrate of the ABCB1 and ABCG2 transporters and it is extruded from certain cancer cells overexpressing ABCB1 and/or ABCG2 transporters, thereby decreasing its efficacy.^[^ ^6a^ ^]^ In contrast, RuZ had a significantly lower binding energy (−5.88 kcal mol^−1^ with ABCG2 and −4.88 kcal mol^−1^ with ABCB1) compared to Dox, suggesting that RuZ has a lower binding affinity for the ABCB1 and ABCG2 transporters and thus, a lower amount is extruded out by the drug‐resistant cancer cells. Furthermore, three self‐assembled anticancer cyclometalated complexes were also docked into the same site and their binding energy was greater than that of RuZ (Figure S14, Supporting Information), suggesting that RuZ had weaker interactions with the two transporters compared to the self‐assembled cyclometalated Pt/Au/Pd compounds.^[^ ^16b‐d^ ^]^ We hypothesize that the higher octahedral geometry, with no extra functional group, largely prevents the significant interaction of RuZ with the active sites of the ABCG2 and ABCB1 transporters, thereby greatly decreasing the likelihood that RuZ levels will be reduced by the overexpression of the ABCB1 and ABCG2 transporters.

Subsequently, we determined the intracellular efflux of RuZ in MDA‐MB‐231/Adr and MDA‐MB‐231 cell lines and the expression of ABCG2 and ABCB1 transporters in MDA‐MB‐231 and MDA‐MB‐231/Adr cells. As shown in Figure S15A,B (Supporting Information), both the expression of the ABCG2 and ABCB1 transporters in the Dox‐resistant MDA‐MB‐231/Adr cells was significantly greater than that in Dox‐sensitive MDA‐MB‐231 cells, indicating that MDA‐MB‐231/Adr cells can extrude certain anticancer drugs to produce MDR via the overexpression of the two ABC transporters. As shown in Figure 2C, ≈64.5% of the normalized intracellular levels of Dox were pumped out in 120 min in MDA‐MB‐231/Adr cells, which is significantly greater than that of MDA‐MB‐231 cells (29.2%), indicating a high level of efflux of Dox. In contrast, the efflux of RuZ from MDA‐MB‐231/Adr cells and MDA‐MB‐231 cells was 14.2% and 9.9%, respectively. These data suggested that the efflux of RuZ in MDA‐MB‐231/Adr cells was not significant and RuZ is not a substrate of the ABCB1 and ABCG2 transporters (Figure 2D) and therefore, high level of RuZ in the cancer cells will be retained, increasing the likelihood of cytotoxic efficacy.

**Figure 3:**
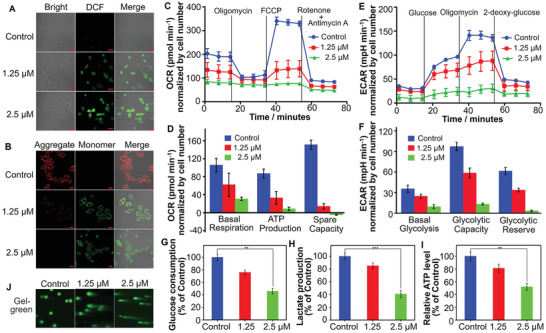
A) DCFH‐DA detection of ROS in MDA‐MB‐231/Adr cells after incubation with RuZ for 24 h. λ_ex/em_ = 485/530 nm. Scale bar: 20 µm. B) JC‐1 detection of mitochondrial dysfunction in MDA‐MB‐231/Adr cells incubated with RuZ for 24 h (λ_ex_ = 485 nm, λ_em/green_ = 530 nm, λ_em/red_ = 590 nm). Scale bar: 20 µm. C) Cellular oxygen consumption in MDA‐MB‐231/Adr cells was detected at 24 h after treatment with RuZ by Seahorse analyzer. Oligomycin (1.5 × 10^−6^ m) was added after 15 min, carbonyl cyanide‐p‐trifluoromethoxyphenylhydrazone (FCCP) (2.0 × 10^−6^ m) was added after 33 min, and rotenone/antimycin A (0.5 × 10^−6^ m) was added after 54 min (*n* = 4, mean ± SD). D) Quantitative comparison of basal respiration, ATP production, and respiratory capacity from (C). E) ECAR was determined in MDA‐MB‐231/Adr cells at 24 h after incubation with RuZ. Glucose (10 × 10^−3^ m) was added after 15 min, oligomycin (1.5 × 10^−6^ m) was added after 33 min and 2‐deoxy‐glucose (50 × 10^−3^ m) was added after 54 min (*n* = 4, mean ± SD). F) Quantitative comparison of basal glycolysis, glycolytic capacity, and glycolytic reserve from (E). G) Glucose consumption over 24 h of incubation with RuZ. (*n* = 4, mean ± SD). *p < 0.05. H) Intracellular lactate production and I) ATP level over 24 h of incubation with RuZ. (*n* = 4, mean ± SD. *p < 0.05. J) Comet assay showing RuZ induced DNA strand breaks in MDA‐MB‐231/Adr cells (λ_ex_ = 488 nm).

Next, we conducted experiments to determine how RuZ decreases cancer cell viability. It has been reported that metal complexes can interact with cellular redox systems and increase the levels of oxidative stress, inducing cancer cell death.^[^ ^26^ ^]^ To determine if redox stress is involved in cancer cell death, we determined the cytotoxicity of RuZ in the presence of N‐acetylcysteine (NAC, an ROS inhibitor) in MDA‐MB‐231/Adr cancer cells. As shown in Figure S16 (Supporting Information), the co‐incubation of NAC with RuZ significantly inhibited the anticancer efficacy of RuZ, suggesting that RuZ alters the levels of ROS. To further ascertain the changes in intracellular oxidative stress produced by RuZ, we determined the cellular ROS levels in MDA‐MB‐231/Adr cells using the ROS‐sensitive probe, DCFH‐DA. The incubation of MDA‐MB‐231/Adr cells with RuZ significantly increased ROS levels (**Figure** **3**A) and these results were further supported by the data obtained from flow cytometry experiments (Figure S17, Supporting Information). The results indicate that the anticancer efficacy of RuZ may be mediated, in part, by increasing the levels of ROS.

H_2_O_2_ is one of the most potent biological oxidizers that mediates cell proliferation, metastasis and MDR.^[^ ^27^ ^]^ The Ru(II) complex can readily interact with H_2_O_2_ to produce the highly toxic ROS, ^•^OH.^[^ ^28^ ^]^ Because the ligand bzp forms a covalent Ru—C bond with Ru, RuZ has a relatively small positive potential in the Ru(III/II) couple, with an E_1/2_([Ru]^3+/2+^) value of +0.66 V (Figure S18, Supporting Information), which is significantly lower than the oxidation potential of H_2_O_2_ (1.77 V), which contributes to the redox interaction between RuZ and H_2_O_2_. Therefore, we determined the redox chemistry between RuZ and H_2_O_2,_ using tetramethylbenzidine hydrochloride (TMB), an ^•^OH indicator that is oxidized to a blue colored product of oxidized TMB, with characteristic absorption peaks at 652 and 900 nm. The TMB solution had an increased absorption at the two peaks in the presence of RuZ, compared to the control group and there was a visible color change from white to blue, indicating the generation of ^•^OH (Figure S19A, Supporting Information). To further confirm the above result, we used electron spin resonance (ESR). The ESR signal of ^•^OH only occurred when RuZ was in the presence of H_2_O_2_ (Figure S19B, Supporting Information), further indicating that RuZ interacts with H_2_O_2_ to produce ^•^OH.

Increased ROS levels have been shown to be positively correlated to the loss of the mitochondrial membrane potential (MMP, ΔΨ_m_) in cancer cells, which can be determined using the MMP‐sensitive probe, JC‐1.^[^ ^29^ ^]^ Following a decrease in MMP, JC‐1 targets mitochondria in a monomeric form that produces green fluorescence in cells. The incubation of MDA‐MB‐231/Adr cells with 1.25 or 2.5 × 10^−6^ m of RuZ produced a clear red to green color shift (Figure 3B), indicating that RuZ decreases the MMP, producing mitochondrial dysfunction. As a result of RuZ‐induced mitochondrial damage, mitochondrial respiration may be inhibited, as indicated by a decrease in the oxygen consumption rate (OCR), as determined using a Seahorse Extracellular Flux analyzer.^[^ ^30^ ^]^ The OCR was decreased in a concentration‐dependent manner in MDA‐MB‐231/Adr cells after incubation with RuZ (Figure 3C). RuZ inhibited mitochondrial respiration and significantly inhibited ATP synthesis. Moreover, 2.5 × 10^−6^ m of RuZ completely inhibited maximal respiration and the spare capacity to affect the transport of protons between mitochondrial membranes, thereby abrogating mitochondrial aerobic respiration (Figure 3D). The decrease in ATP production could further overcome MDR mediated by the ABCB1 and ABCG2 transporters as they require ATP to extrude drugs from cells.

Mitochondrial aerobic respiration is an important energy‐producing system in cells, whereas most cancer cells primarily dependent on glycolysis (the Warburg effect), which can induce MDR to anticancer drugs.^[^ ^31^ ^]^ Consequently, we determined the glycolytic capacity of MDA‐MB‐231/Adr cancer cells by measuring the extracellular acidification rate (ECAR) that is based on monitoring the change in pH. RuZ, at 2.5 × 10^−6^ m, significantly decreased the ECAR and completely inhibited glycolysis (Figure 3E). Furthermore, RuZ not only inhibited basal glycolysis but also prevented glycolysis by suppressing the glycolytic capacity (Figure 3F). Thus, the inhibition of OCR and ECAR significantly inhibited mitochondrial respiration and glycolysis, which are the major processes involved in producing ATP and other molecules required for cellular viability. Glucose utilization and lactate production were significantly reduced in MDA‐MB‐231/Adr cancer cells after incubation with RuZ, compared to cells incubated with vehicle (Figure 3G,H), further indicating a suppression in mitochondrial respiration and glycolysis. These results indicate that in vitro, RuZ decreases ATP levels by inhibiting mitochondrial respiration and glycolysis and this is reflected by the reduction in the intracellular levels of ATP in MDA‐MB‐231/Adr cells (Figure 3I). It is possible that the decrease in intracellular ATP levels could decrease the efflux activity of the ABCB1 and ABCG2 transporters and overcome MDR due to nutritional deprivation. Overall, the significant intracellular retention and anticancer efficacy of RuZ could be due, in part, to the inhibition of mitochondrial respiration and glycolysis.

**Figure 4:**
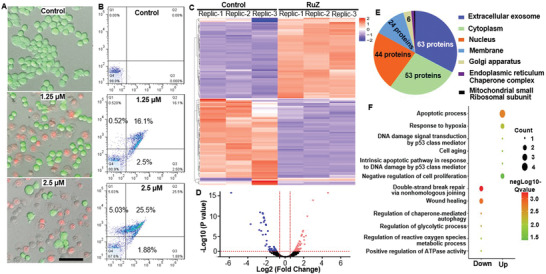
A) Calcein AM/PI detection of cell death in MDA‐MB‐231/Adr cells incubated with RuZ for 24 h (Calcein AM: λ_ex_ = 488 nm, λ_em/green_ = 550 nm; PI: λ_ex_ = 525 nm, λ_em/red_ = 630 nm). Scale bar: 100 µm. B) The flow cytometry results of cell death treated RuZ for 24 h by Annexin V‐FITC/PI assay (FITC: λ_ex_ = 488, λ_em/green_ = 525 nm; PI: λ_ex_ = 525 nm, λ_em/red_ = 630 nm). C) Heatmap cluster of proteomic changes before and after RuZ treatment for 24 h in MDA‐MB‐231/Adr cells. D) Volcanic map of proteomic changes before or after RuZ treatment for 24 h. The vertical pink dotted lines indicate the cut‐off of log2 fold change (1 or −1). The horizontal pink dotted line indicates the cut‐off of p‐value (0.05). E) Subcellular localization of proteins that has differentially expression after RuZ treatment for 24 h. F) Gene Ontology (GO) analysis of changed proteins after RuZ treatment for 24 h.

It has been reported that oxidative stress and metabolic inhibition can produce cellular DNA damage.^[^ ^32^ ^]^ Since RuZ targets mitochondria and nuclei, we determined its effect on the integrity of DNA in MDA‐MB‐231/Adr cells, using a single cell gel. There was no DNA strand breakage in the cells incubated with vehicle (Figure 3J). In contrast, the incubation of MDA‐MB‐231/Adr cells with 1.25 or 2.5 × 10^−6^ m of RuZ produced DNA that formed a “comet” shape around the nucleus, indicating the presence of DNA damage. Subsequently, RuZ, after 24 h of incubation, produced a concentration‐dependent increase in apoptosis, as determined using the calcein AM/PI assay, in MDA‐MB‐231/Adr cells (**Figure** **4**A,B). Furthermore, 2.5 × 10^−6^ m of RuZ induced a small increase in cellular necrosis. Our results suggest that RuZ produces effective cytotoxicity by multiple mechanisms that contribute to overcoming MDR mediated by the overexpression of the ABCB1 or ABCG2 in MDA‐MB‐231/Adr cells.

To further determine the mechanism of action of RuZ, we conducted proteomic profiling experiments. The incubation of MDA‐MB‐231/Adr cells with 2.5 × 10^−6^ m of RuZ, altered the expression of proteins based on the criteria of a 1.5‐fold change in protein expression, compared to cells incubated with vehicle (Figure 4C). Specifically, 59 proteins were upregulated and 57 proteins were downregulated (Figure 4D). Among these proteins, 54% were in exosomes, 45% in the cytoplasm and 37% in the nucleus (Figure 4E). Based on the Gene Ontology (GO) results (Figure 4F), glycolysis, DNA repair, ROS metabolism and ATPase proteins, were significantly downregulated in MDA‐MB‐231 cells. Furthermore, apoptosis, DNA damage and anti‐proliferation‐related proteins were significantly increased in MDA‐MB‐231/Adr cells after incubation with 2.5 × 10^−6^ m of RuZ and it is likely that these effects contributed to an increased likelihood of cancer cell death.

We also determined the biodistribution and metabolism of RuZ, as these data will help in establishing a preclinical profile for RuZ. As shown in Figure S20 (Supporting Information), the maximum level of RuZ at the tumor site occurred 24 h after the intravenous administration of 3.0 mg kg^−1^ of RuZ. The blood circulation profile of RuZ is shown in Figure S21 (Supporting Information) and the half‐life of RuZ was 5.69 h. However, only a relatively low amount of RuZ accumulated in tumors, suggesting that RuZ does not efficiently target tumor‐bearing tissues. RuZ was primarily metabolized by the liver and was sequestered in the spleen (Figure S20, Supporting Information). In addition, to determine the clearance of RuZ in mice, we measured the levels of ruthenium in the urine and feces at different time points up to 96 h. As shown in Figure S22 (Supporting Information), ruthenium was detected in the urine and feces, suggesting that RuZ was excreted in the urine and feces. The acute overt toxicity of RuZ was determined by monitoring the weight of healthy mice following the i.v. administration of 3.0, 6.0, or 12 mg kg^−1^ of RuZ for 10 days (Figure S23, Supporting Information). There was no significant difference in the body weight of mice treated with RuZ compared to mice treated with the vehicle. We also determined the effect of 3.0 mg kg^−1^ of RuZ on: 1) the plasma levels of parameters indicative of blood chemistry and 2) indices of blood biochemistry. As shown in Figures S24 and S25 (Supporting Information), the i.v. administration of RuZ to mice did not significantly alter any of the indices measured compared to mice treated with i.v. PBS. Furthermore, RuZ (40 µg mL^−1^) did not cause hemolysis of red blood cells (Figure S26, Supporting Information). Overall, our results in mice suggest that at the doses administered, RuZ had a favorable in vivo toxicity profile.

**Figure 5:**
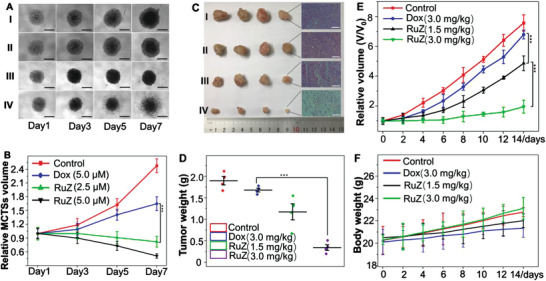
A) The effect of Dox and RuZ on the growth of MDA‐MB‐231/Adr MCTSs, I: control; II: 5.0 × 10^−6^ m of Dox; III: 2.5 × 10^−6^ m of RuZ; IV: 5.0 × 10^−6^ m of RuZ. Scale bar: 300 µm. B) The corresponding relative volume changes (*V*/*V*_0_) come from A (*n* = 3, mean ± SD. ***p < 0.0004. C) Digital photos of excised MDA‐MB‐231/Adr tumors and the representative H&E stained tumor slices from mice at day 14 after different peri‐tumor treatments, I: control; II: 3.0 mg kg^−1^ of Dox; III: 1.5 mg kg^−1^ of RuZ; IV: 3.0 mg kg^−1^ RuZ. Scale bar: 125 µm. D) Tumor weights of MDA‐MB‐231/Adr‐bearing mice at the end of the 14‐day treatment period. E) Tumor growth curves ***p < 0.0005. F) The effect of Dox and RuZ on the body weight of mice with xenografted tumors.

Based on our significant in vitro results, we determined the efficacy of RuZ was in 3D multicellular tumor spheroids (MCTSs) and in a tumor‐bearing mouse model. As shown in **Figure** **5**A,B, 5.0 × 10^−6^ m of Dox did not significantly alter the growth of MDA‐MB‐231/Adr MCSTs on days 1, 3 or 5, compared to the control (phosphate buffered saline treated) group, although there was a significant decrease on day 7. In contrast, the growth of the MCTSs was significantly decreased by 2.5 or 5.0 × 10^−6^ m of RuZ, compared to the control and the 5.0 × 10^−6^ m Dox groups. Thus, RuZ was efficacious in inhibiting MDA‐MB‐231/Adr cell proliferation. Next, we used MDA‐MB‐231/Adr cells to create xenografts in mice and determined the efficacy of the peritumoral injection of RuZ (Figure 5C–E). There was a rapid and progressive growth of tumors in the control and Dox (3.0 mg kg^−1^) groups (Figure 5E), indicating that MDA‐MB‐231/Adr cells can proliferate rapidly and that MDA‐MB‐231/Adr tumors are resistant to Dox. In contrast, 1.5 mg kg^−1^ of RuZ significantly inhibited tumor growth (maximal inhibition of about 33% on Day 14) compared to Dox. Furthermore, 3.0 mg kg^−1^ of RuZ produced a greater inhibition of tumor growth (maximal inhibition of 62% on Day 14), compared to mice treated with Dox and 1.5 mg kg^−1^ of RuZ, which was only about one‐ninth the weight of the control group tumors (Figure 5D,E). The peritumoral injection of either 1.5 or 3.0 mg kg^−1^ of RuZ did not significantly alter the body weight of the mice compared to those treated with vehicle (Figure 5F). Furthermore, RuZ (1.5 or 3.0 mg kg^−1^) did not significantly alter the morphology of the heart, liver, spleen, lung, kidney and intestines (Figure S27, Supporting Information). Therefore, at the doses used in this study, RuZ did not produce significant systematic toxicity in mice.

## 3 Conclusion

We have developed a simple, versatile and efficacious self‐assembled cyclometalated complex, RuZ, to overcome MDR cancer cells. RuZ spontaneously self‐assembles into nanoaggregates in an aqueous medium by directed self‐assembly, in the absence of an additional reagent. Due to the high drug‐loading rate of the self‐assembly and low affinity of RuZ for the ABCB1 and ABCG2 transporters, a high intracellular level of RuZ was retained in the MDR cancer cells. Importantly, RuZ significantly inhibited mitochondrial respiration and oxygen glycolysis in MDA‐MB‐231/Adr cells, which markedly decreased intracellular ATP levels and resulted in the inactivity of efflux pumps, thereby increasing the retention of RuZ and thus, the likelihood of cell death. Furthermore, RuZ significantly increased the level of ROS and DNA damage, which increased apoptotic‐induced cancer cell death. Finally, in vivo, RuZ had a favorable biosafety profile and was efficacious in inhibiting the proliferation of Dox‐resistant tumors in mice xenografted with MDA‐MB‐231/Adr cells. These results, provided they can be extrapolated to humans, suggests that RuZ may represent an efficacious treatment for cancers that are MDR.

## Conflict of Interest

The authors declare no conflict of interest.

## Supporting information

Supporting Information

Supporting Information

## Data Availability

Research data are not shared.
