# Supplementary material for: Cycloruthenated Self‐Assembly with Metabolic Inhibition to Efficiently Overcome Multidrug Resistance in Cancers
Source: Adv Mater. 2021 Oct 24;34(1):2100245. doi: 10.1002/adma.202100245 (PMC11468970; doi:10.1002/adma.202100245)
Supplement: Supplementary file 1 — Supporting Information [file ADMA-34-2100245-s001.pdf]

ADVANCED MATERIALS

Supporting Information

for *Adv. Mater.,* DOI: 10.1002/adma.202100245

Cycloruthenated Self-Assembly with Metabolic Inhibition to Efficiently Overcome Multidrug Resistance in Cancers

Jia Li, Leli Zeng, Zheng Wang, Hengxing Chen, Shuo Fang, Jinquan Wang, Chao-Yun Cai, Enming Xing, Xinxing Liao, Zhi-Wei Li, Charles R. AshbyJr, Zhe-Sheng Chen,* Hui Chao,* and Yihang Pan**

© 2021 Wiley-VCH GmbH

Supporting Information

Cycloruthenated Self-Assembly with Metabolic Inhibition to Efficiently Overcome Multidrug Resistance in Cancers

Jia Li[†][a][b], Leli Zeng[†][a][b][c], Zheng Wang[d], Hengxing Chen[a], Shuo Fang[a], Jinquan Wang[d], Chao-Yun Cai[c], Enming Xing[a], Xinxing Liao[b], Zhi-Wei Li[b], Charles R. Ashby Jr[c], Zhe-Sheng Chen*[c], Hui Chao*[a][b], and Yihang Pan*[a]*

[a] Guangdong Provincial Key Laboratory of Digestive Cancer Research, Precision Medicine Center, The Seventh Affiliated Hospital, Sun Yat-Sen University, Shenzhen 518107, Guangdong, P. R. China

[b]MOE Key Laboratory of Bioinorganic and Synthetic Chemistry, School of Chemistry, Sun Yat-Sen University, Guangzhou 510275, P. R. China

[c]College of Pharmacy and Health Sciences, St. John's University, New York, NY 11439, USA

[d]College of Chemistry and Chemical Engineering, Key Laboratory of Chemical Additives for China National Light Industry, Shaanxi University of Science and Technology, Xi'an 710021, P. R. China

[e]Guangdong Province Key Laboratory for Biotechnology Drug Candidates, School of Bioscience and Biopharmaceutics, Guangdong Pharmaceutical University, Guangzhou, P. R. China

[†]These authors contributed equally.

*Correspondence: E-mail: zenglli6@mail.sysu.edu.cn; chenz@stjohns.edu; ceschh@mail.sysu.edu.cn; panyih@mail.sysu.edu.cn.

Table of Contents

Experimental Section

Materials and instruments

Synthesis and characterization of RuZ

Spectroscopic measurements

Electron spin resonance measurements

HPLC measurements

TEM and SEM measurements

Single-Crystal X-ray crystallography

ABCG2 and ABCB1 induced-fit docking

Self-assembly simulation

Cell culturing

Cytotoxicity assay

Cell uptake studies

Organelle distribution studies

Intracellular ATP detection

ROS generation assay

Cyclic voltammetric measurements

Induction of mitochondrial dysfunction

Comet assay

Protein analysis

MCTSs detection

Western blot analysis

Proteomic profiling analysis

Seahorse assay

***In vivo* antitumor performance**

***In vivo* pharmacokinetics and biodistribution**

Hemolysis test

Blood biochemistry and determination of hematological parameters

Excretion study

Histological examination

Supporting Figures and Tables

Figure S1 The representative self-assembled cyclometalated complexes

Figure S2 ESI-MS spectrum of RuZ

Figure S3 HRMS spectrum of RuZ

Figure S4 HPLC trace for complex RuZ

Figure S5 ^{1}H NMR spectra of RuZ in CD_3CN

Figure S6 ^{13}C NMR spectra of RuZ in CD_3CN

Figure S7 The spectra of absorption and emission of RuZ

Figure S8 The stacking image of RuZ molecules

Figure S9 2D DOSY NMR spectra of RuZ in CD_3CN

Figure S10 Digital photos and Tyndall effect of RuZ in DI water

Figure S11 Hydrodynamic radius of the RuZ nanoparticles in DI water and PBS

Figure S12 The cytotoxicity of RuZ in various cancer cell lines

Figure S13 The uptake of different compounds in various cancer cells

Figure S14 The docked positions of the drugs and proteins for ABCG2 and ABCB1

Figure S15 The effect of RuZ on the expression of ABCG2 and ABCB1

Figure S16 The effect of N-acetylcysteine on the cytotoxicity of RuZ

Figure S17 Analysis of intracellular ROS levels

Figure S18 Cyclic voltammogram of RuZ in MeCN

Figure S19 Analysis of hydroxyl radical levels in aqueous solution

Figure S20 Ruthenium in main organs and tumors

Figure S21 Blood circulation curve of RuZ

Figure S22 Analysis of the RuZ levels in the urine and feces

Figure S23 Acute toxicity experiments with RuZ

Figure S24 Hematological analysis

Figure S25 Blood biochemical analysis

Figure S26 Hemolysis analysis

Figure S27 Hematoxylin and eosin images of tissues from mice treated with RuZ

Table S1 Toxicity of complexes in normal cells

Table S2 Toxicity of several clinical drugs in cancer cells

Table S3 The crystallography data and structure refinement for RuZ

Table S4 The bond lengths for RuZ

Author Contributions

References

Experimental Procedures

Materials and instruments

Unless otherwise indicated, all of the reagents were purchased from commercial sources and used without further purification. Electrospray ionization mass spectra (ESI-MS) was recorded using a LCQ system (Finnigan MAT, USA). High resolution mass spectra (HRMS) was recorded using Orbitrap LC/MS. Microanalyses (C, H, and N) were carried out using an Elemental Vario EL CHNS analyzer (Germany). The ^{1}H NMR, ^{13}C NMR and 2D DOSY NMR spectra were recorded were recorded on Varian Mercury Plus 400 Nuclear Magnetic Resonance Spectrometer. Confocal cell imaging was conducted using a LSM 810 (Carl Zeiss, Germany) Laser Scanning Confocal Microscope. The ultraviolet-visible-NIR absorption spectra and fluorescence (FL) spectra of the samples were determined using a Shimadzu 2450 UV-vis spectrophotometer and a RF-5301PC fluorescence spectrometer (SHIMADZU, Japan), respectively. Flow cytometry experiments were conducted using a BD FACS CantoII. Transmission electron microscopy (TEM) investigation was carried out on a FEI Tecnai G2 F30. Scanning electron microscope (SEM) inverstigations were carried out on a GeminiSEM500. Dynamic light scattering (DLS) was carried out using a EliteSizer instrument at room temperature. All of the compounds were dissolved in DMSO just before the experiments and the final DMSO concentration was less than 1% (v/v). Ruthenium chloride hydrate, Benzo[*h*]quinoline (bzq, M: 179.22), 1,10-phenanthroline (phen, M: 180.21), dipyrido[3,2-*a*:2',3'-*c*]phenazine (dppz, M: 282.31), doxorubicin, mitoxantrone and cisplatin were purchased from Sigma-Aldrich (Shanghai, China). 3-(4,5-dimethylthiazol-2-yl)-2,5-diphenyltetrazolium bromide (MTT) was purchased from Alfa Aesar (Shanghai, China). The 2’,7’-dichlorofluorescin diacetate (DCFH-DA) kit, the Annexin V-FITC apoptosis assay kit and the bicinchoninic acid (BCA) protein sssay kit were obtained from the Beyotime Institute of Biotechnology (China). 5′,6,6′-tetrachloro-1,1′,3,3′-tetraethylbenzimidazolyl-Carbocyanine iodide (JC-1), propidium iodide (PI) and PicoGreen were purchased from Yeasen (Shanghai, China). The ABCG2 and ABCB1 antibodies were purchased from abcam.

Synthesis and characterization of RuZ

The synthetic route of RuZ is shown in Scheme S1. $[Ru(C_6H_6)Cl_2]_2$ (500 μmol) and bzq (500 μmol) were added into a MeCN solution (25 mL) and stirred at 45°C for 24 h. After an anion exchange with NH_4PF_6, there was a separation of the yellow mixed solution $[Ru(bzq)(CH_3CN)_4]PF_6$. Next, phen (500 μmol) was added to the yellow solution and the mixture was stirred at room temperature for 12 h, yielding $[Ru(bzq)(phen)(CH_3CN)_2]PF_6$, that was purified by column chromatography using CH_3CN-CH_2Cl_2 (1:8, v/v) as the eluent. Finally, the ligand, dppz (500 μmol), and $[Ru(bzq)(phen)(CH_3CN)_2]PF_6$, were refluxed in 15 mL N,N-dimethylformamide (DMF) for 24 h and the solvent was evaporated under a vacuum, producing a dark residue. The dark residue was dissolved in dichloromethane (5 mL) and the solution was purified with aluminium trioxide using a CH_3CN/toluene (1:8 to 8:1, v/v) eluent. The purple pure product, RuZ, was collected. The recrystallization of RuZ was also obtained by vapor diffusion from diethyl ether to a $MeCN/CH_2Cl_2$ solution in presence of KPF_6. (Yield: 177.4 mg, 202 μmol, 40%). Anal. ESI-MS (m/z). 742 $(M\text{-}PF_6)^+$. Calcd for $C_{43}H_{26}N_7F_6RuP$ (%): C, 58.17; H, 2.93; N, 11.05. Found (%): C, 58.22; H, 3.04; N, 11.01. ^{1}H NMR: (400 MHz, CD_3CN) δ = 9.47 (d, *J*=8.1, 1H), 9.25 (d, *J*=8.1, 1H), 8.42 – 8.29 (m, 6H), 8.22 (d, *J*=8.2, 2H), 8.14 – 8.03 (m, 6H), 7.89 (d, *J*=8.8, 1H), 7.75 (d, *J*=8.4, 2H), 7.56 – 7.50 (m, 1H), 7.49 – 7.43 (m, 2H), 7.37 (d, *J*=7.5, 1H), 7.29 – 7.23 (m, 1H), 7.16 (t, *J*=7.4, 1H), 6.52 (d, *J*=6.4, 1H). ^{13}C NMR: (400 MHz, CD_3CN) δ = 156.43, 156.10, 151.46, 151.29, 150.75, 150.58, 149.97, 148.87, 147.94, 147.40， 142.17, 141.42, 139.99. 134.29, 133.55,

132.80, 132.65, 131.61, 130.02, 129.18, 127.83, 126.27, 125.81, 124.62, 123.45, 123.41, 121.27, 118.80.

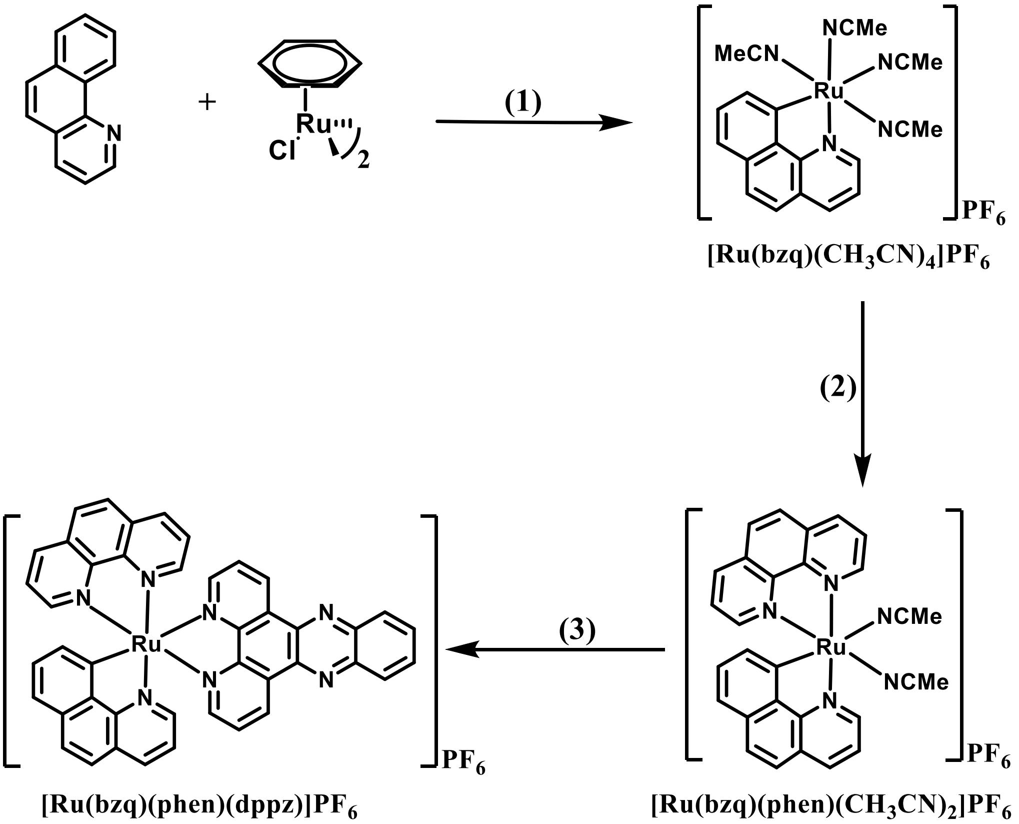

Scheme S1. Synthetic scheme of Ru(II) complex, RuZ. Reaction conditions: (1) KPF_6, MeCN, reflux, 24 h. (2) phen, MeCN, reflux, room temperature, 12 h. (3) dppz, DMF, reflux, 12 h.

Spectroscopic measurements

The spectra of TMB were performed using the following method: A solution of TMB (10 mmol/L) and H_2O_2 (0.5 mM), in the presence or absence of RuZ (10 µM), were allowed to sit for 1 h and analysis was done using a Shimadzu 2450 UV-vis spectrophotometer.

Electron spin resonance measurements

The ESR spectra were recorded using a Bruker Model A300 spectrometer (20 mW microwave power, 100 G-scan range, and 1 G field modulation, rt). Three samples: free RuZ, free H_2O_2, RuZ and H_2O_2 (H_2O_2: 0.5 mM, RuZ: 10 µM) were dissolved in aerated methanol containing 10 µL of 5,5-Dimethyl-1-pyrroline N-oxide (DMPO) as a •OH spin trap and siphoned into capillary tubes in the dark. The ESR spectra of the sample was measured in the dark.

HPLC measurements

A methanol solution of RuZ was injected into an HPLC system (Thermo, USA) connected to a UV/Vis spectrophotometer. A Hypersil Gold Dim (Thermo, USA) reversed-phase column was used, with a flow rate of 0.5-2 mL/min. The HPLC runs were performed using a linear gradient of B (0.1% TFA in acetonitrile; HPLC grade) in A (distilled water containing 0.02% trifluoroacetic acid (TFA) and 0.05% HCOOH). The absorption spectra at 370 nm excitation was determined.

TEM and SEM measurements
Ten uL of RuZ DMSO solution (10 mM) was added into 1 mL of DI water and the solution was blown with a pipette. Notion: The nano-scale self-assembly was formed immediately and used for TEM and SEM. Note: in order to confirm the repeatability of data, the formed nanoparticles (10 μL of 10 mM RuZ DMSO solution was added into 1 mL of DI water) were used without further teatment. For comparison, the nanoparticles were added into the DMEM conditions for further testing after centrifugation at 12,000 rpm for 15 min.

Single-Crystal X-ray Crystallography
Single crystals of $C_{43}H_{26}F_6N_7PRu$ [RuZ] were obtained by vapor diffusion from diethyl ether to a $MeCN/CH_2Cl_2$ solution. A suitable crystal was selected on a diffractometer. The crystal was kept at 150 K during data collection. Using Olex2[1], the structure was solved with the olex2.solve structure solution program using Charge Flipping and refined with the ShelXL refinement package using Least Squares minimization[2].

ABCG2 and ABCB1 induced-fit docking and molecular dynamics simulation
Human ABCG2 (PDB Code: 6FFC) and ABCB1 (PDB Code: 6FN1) crystal structures were downloaded and prepared by AutodockTools4 program, and transmembrane domain (TMD) of both proteins were defined as docking grids using AutoGrid4.[3] Geometry optimization of all the compounds was performed by Gaussian software, and optimized structures were docked into the abovementioned grids using the Autodock4 with default settings.

Self-assembly simulation
The Ru-coordinated compound was constructed by Gaussview, and all structures [SJ Wu1] were optimized at the level of M06-2X functional theory (Non-metallic heavy atoms were optimized by B3LYP/6-31G* module, and Ru atom was optimized by SDD module) by Gaussian09. Bonded model for molecular dynamic simulation was built up using the MCPB.py module in the Amber18 molecular dynamics package, and the dodecamer was constructed by tleap module. The dodecamer complex was solvated in a 12 Å*12 Å*12 Å TIP3P water box. The position of water molecules was optimized by 400 steps of steepest descent minimization followed by 3,600 steps of conjugate gradient minimization, and the whole system was optimized by 8,000 steps of conjugate gradient minimization. Then 200 ps (100,000 steps) simulation was used to heat the system up to 300 K, which followed by 500 ps (250,000 steps) simulation to raise the pressure to 1 bar. After a 50 ps equilibration run under 300 K and 1 bar, 150 ns molecular dynamic simulation was taken by Pmemd.cuda module in Amber18.

Cell culturing
All cell lines (the non-resistant cancer cell lines, the drug-resistant cancer cell lines and the normal cell lines) were cultured in DMEM/1640 supplemented with 10% fetal bovine serum (FBS) and 50 U/mL streptomycin and 50 ng/mL penicillin (Gibco BRL) in a humidified incubator under 5% CO_2 and 20% O_2 at 37 °C. All the drug-resistant cells were grown in drug-free culture medium for more than two weeks before assay.

The colchicine-selected ABCB1-overexpressing KB-C2 cells were cultured with 1 µg/mL of colchicine. The KB/ATO cells were cultured with 2 μM of As_2O_3. The doxorubicin-selected, ABCB1-overexpressing SW620/AD300 cells were cultured with 30 nM of doxorubicin. The hepatoma carcinoma cell line, BEL-7404/CP20 (7404/CP20) and BIU-87/DDP, was cultured with 0.2-1.0 µM of cisplatin. The mitoxantrone-selected, ABCG2-overexpressing H460/MX20 lung cancer cells were cultured with 20 nM of mitoxantrone. The doxorubicin-selected, ABCB1-overexpressing MDA-MB-231/Adr triple negative breast cancer cells were cultured with 30 nM of Dox

Cytotoxicity assay

The cytotoxicity of the drugs was determined using the MTT assay and were tested using the half dilution method. The highest concentration of the RuZ was 100 μM (10 μL of 10 mM RuZ DMSO solution was added into 1 mL sterile distilled water). Approximately 10,000 cells were seeded into each well in a 96-well plate and grown overnight. Subsequently, the old culture medium was replaced by 200 μL of the cell culture medium with different concentrations of the drugs. The plates were incubated for 72 h. Fifteen μL of the MTT solution (5 mg/mL) was added to each well. After 4 h of incubation, the culture medium was removed and 150 μL of DMSO was added to each well. The optical density of each well was recorded using a microplate spectrophotometer at a wavelength of 595 nm.

Cell drug uptake studies

The cells were plated at a density of 10^6 cells/mL in 10 mL of DMEM for 24 h, and either 2.5 μM of RuZ, As_2O_3 or cisplatin were added to the culture medium and incubated for 2 h. The culture medium was removed, and the cells were washed three times with cold PBS and were collected in a tube and digested in a solution of 15% HNO_3 (1 mL) and 10% H_2O_2 (1 mL) for 72 h. Each sample was diluted with 5 mL of Milli-Q water. The content of Ru, Pt or As was determined using inductively coupled plasma mass spectrometry (ICP-MS). The intracellular levels of mitoxantrone (1 μM, 2 h incubation period) were determined using the following method: the lysed sample containing [^{3}H]-mitoxantrone was placed in scintillation vials containing 5 mL of liquid scintillation cocktail and the radioactivity was determined using a Packard TRI-CARB 1900CA liquid scintillation analyzer (Packard Instrument, Downers Grove, IL), as previously described.[4] The intracellular level of the doxorubicin (1 μM, 2 h incubation period) was determined by measuring the fluorescence of the culture medium (λ_{ex} = 480 nm, λ_{em} = 580 nm) to obtain the relative value.

Organelle distribution studies

MDA-MB-231/Adr cells were incubated with RuZ (2.5 μM) at 37 °C for 2 h (1 × 10^6 cells). The medium was removed and the cells were washed twice with 10 mL PBS, and digested with trypsin and counted. All of the cells were equally divided into two parts and processed using a Nucleus Extraction Kit and Cytoplasmic and Mitochondrial Protein Extraction Kit, as per the manufacturer's instructions. The cell extracts were digested using 68% HNO_3 (rt, 48 h) and the level of ruthenium was determined using ICP-MS.

Intracellular ATP detection[5]

MDA-MB-231/Adr cells (5 × 10^3/well) were seeded into white 96-well culture plates (Corning) for 24 h and incubated with different concentrations of RuZ at 37°C for 24 h. One hundred microliters of the reagent from the CellTiter-Glo® Luminescent Cell Viability Assay kit was added and the samples processed according to the manufacturer’s instructions (G7570, Promega, USA). The intracellular content of ATP was determined using a multifunctional reader that measured chemoluminescence (TECAN Infinite M200 PRO). The ATP levels for the control group (i.e., untreated samples) was considered to be 100%, so that the other groups were compared to the control group and the cell viability was calculated for each group.

ROS generation assay

MDA-MB-231/Adr cells were incubated with RuZ (1.25 or 2.5 μM) for 24 h and were trypsinized, collected and stained with DCFH-DA according to the manufacturer’s instructions. The DCF dye was excited at 480 nm, with a green fluorescence emission (530 nm). The results were obtained using flow cytometry (BD FACS CantoII).

Cyclic voltammetric measurements
Cyclic voltammetry was done in anhydrous acetonitrile with 0.1 M of tetra-n-butylammonium hexafluorophosphate, $[Bu_4N][PF_6]$, under nitrogen. The working electrode was a BAS Pt disk electrode, the auxiliary electrode was a Pt wire and the reference electrode was Ag/AgCl.

Induction of mitochondrial dysfunction
The mitochondrial transmembrane potential was assessed using the reagent, JC-1. MDA-MB-231/Adr cells (5×10^4 cells/mL, 2 mL, single cell) were cultured on to 35 mm confocal dishes for 24 h and then incubated with RuZ (1.25 and 2.5 μM) for 24 h, followed by staining with JC-1 (5 μg/mL, 30 min). The cells were washed twice with 2 mL of PBS and changes in the mitochondrial membrane potential were measured using confocal laser scanning microscopy.

DNA damage assay[6]
MDA-MB-231/Adr cells were incubated with RuZ (1.25 or 2.5 μM) in 6-well plates for 24 h. The cells were digested with trypsin and collected. The cells were combined with molten low-melting point agarose at a ratio of 1:10 (v/v), and the low melting-point agarose was then spread on slides precoated with 1% normal-melting agarose, and the slides were placed in the refrigerator at 4°C for 30 min. The slides were immersed in a prechilled lysis solution (2.5 M NaCl, 100 mM EDTA, 10 mM Tris base, 1% Triton X-100, and 10% DMSO, pH 10) and placed back in the refrigerator for approximately 30 min. The lysis buffer was removed, and the slides were immersed in an alkaline unwinding solution for 20 min in the dark. The slides were removed from the alkaline unwinding solution and electrophoresed at 21 V and 300 mA for 30 min in the dark. After electrophoresis, the slides were washed two times in ultrapure water for 5 min each and immersed in 35 mL of 100% ethanol for 5 min. Finally, the slides were allowed to dry for 5 min before staining using a PI solution (2 μg/ mL) for 15 min in the dark and analysed using an inverted fluorescence microscope (Zeiss Axio Observer Z1, Germany).

Protein detection
MDA-MB-231/Adr cells (5×10^6) were seeded into 10 cm tissue culture dishes overnight and incubated with RuZ (1.25 or 2.5 μM) for 24 h. The cells were washed twice with precooled PBS and digested with trypsin, and lysed in a radioimmune precipitation assay buffer (RIPA) containing a protease inhibitor cocktail (Blue Skies) for 45 min on ice. The lysates were centrifuged at 15,000 rpm for 15 min at 4 °C, and the protein concentrations were determined using the BCA protein assay.

MCTSs detection[7]
Two hundred μL of culture media, containing 5000 MDA-MB-231/Adr cells, was transferred to 0.95% agarose-coated transparent 96-well plates. The cells formed MCTS aggregates that were approximately 400 μm in diameter after three days of incubation. One hundred microliters of the old medium in the cultured MCTSs was replaced with either drug-supplemented or drug-free standard culture media. MCTSs were imaged using a phase contrast microscope (Zeiss Axio Observer D1, Germany) under a 10×objective to evaluate their integrity and diameter every two days. The culture media of all cells was refreshed every two days. The images and diameter data of MCTSs were collected every two days. The volume of the MCTSs was determined using the following equation: $4/3 \times \pi \times (\text{diameter}/2)^3$.

Western blot analysis
One million MDA-MB-231 or MDA-MB-231/Adr cancer cells were collected and were washed with ice-cold PBS and lysed using a radio immune precipitation assay (RIPA) buffer

for 30 min on ice. The lysates were centrifuged at 15,000 rpm at 4°C and the supernatant was collected. The protein concentration of each samples was tested using a BCA protein assay reagent kit. The protein samples were separated on SDS-polyacrylamide gel electrophoresis and transferred to PVDF membranes in an ice bath. The membrane was submerged in a blocking reagent for 2 h, washed, and incubated with the primary antibodies (Anti-BCRP/ABCG2, Anti-p-glycoprotein, abcam) TBST solution overnight at 4°C. After washing with TBST, the membrane was incubated with the secondary antibody. The signal was detected using enhanced chemiluminescence and exposure of Xray film.

Proteomic profiling analysis

MDA-MB-231/adr cells were incubated with either vehicle (PBS) or 2.5 μM of RuZ for 24 h. Cells were then lysed with NETN buffer (1% protease inhibitor cocktail) for 30 min. The supernatant was removed by centrifugation at 15,000 g for 5 min at room temperature to remove debris. The protein was enzymatically hydrolyzed and precipitated with with acetone. In brief, 300 μg of protein was diluted with 100 μL of 50 mM NH_4HCO_3, followed by the addition of 400 μL of pre-cooled acetone. The tubes were incubated overnight at -20°C and the next day, the tubes were centrifuged at 15000 g for 30 min at 4°C. The precipitate was washed once with 70% ethanol and the pellet was freeze-dried. The pellet was resuspended in UA (8 M urea, 150 mM Tris-HCl pH 8.0) buffer. Next, 2 μL of 2 mM dithiothreitol (DTT) was added and the samples incubated for 1.5 h at 30 °C. Thirteen μL of 10 mM of iodoacetamide (IAA) was added and the samples were allowed to sitfor 45 min at room temperature in the dark. The sample was diluted by adding 50 mM of NH_4HCO_3. Finally, trypsin was added, at a 1:50 trypsin-to-protein mass ratio for 18 h. A 10% solution of trifluoroacetic acid was used to terminate the enzymatic hydrolysis reaction. The amount of protein was determined using the Pierce Quantitative Colorimetric Peptide Assay kit, as per the manufacturer's instructions. We used 1 μg of protein from each of the two groups for the LC-MS/MS analysis. We used the DAVID database (https://david.ncifcrf.gov/) to predict the subcellular localization of the affected proteins. Gene Ontology (GO) analysis, based on the DAVID database, were constructed using the ClueGO unit. The GO process with a corrected p-value < 0.05 was considered statistically significant.

Seahorse assay

The effect of RuZ on bioenergetics was determined using the Seahorse XF glycolysis assay (Agilent Technologies, Berlin, Germany), according to the manufacturer's instructions. Prior to the assay, XF sensor cartridges were hydrated based on the instructions from the manufacturer. One mL of Seahorse Bioscience calibrant was added to each well of an XF utility plate, and the XF sensor cartridges were placed on top of the utility plate and kept in a 37°C incubator without CO_2 for 12 h. A total of 2×10^4 MDA-MB-231/Adr cells/well were seeded in 96-well plates, followed by culturing at 37°C for 24 h, under a 5% CO_2 atmosphere. The cells were incubated with RuZ (1.25 or 2.5 μM) for 24 h and each well was washed twice with XF assay medium (Seahorse Bioscience). Subsequently, the XF assay medium (pH 7.4), containing 25 mM of glucose, 1 mM of pyruvate, and 2 mM of glutamine (Sigma-Aldrich) were added to the wells, along with the test compounds, and the cells were equilibrated at 37°C in a CO_2-free incubator for 45 min. The oxygen consumption rate (OCR) was measured following the addition of the electron transport inhibitors, oligomycin (1.5 μM)), FCCP 0.5 μM), rotenone (1 μM) and antimycin A (1 μM) were added 15, 33 and 54 min, respectively.Parameters of respiration, basal respiration, coupling efficiency spare respiratory capacity and the extracellular acidification rate (ECAR) were measured simultaneously to establish a baseline measurement. Glycolysis, glycolytic capacity, glycolytic reserve and non-glycolytic acidification were calculated by subtracting the average rates before and after the addition of glucose (10 mM), oligomycin (1.5 μM) and 2-deoxy-d-glucose (2-DG)

(50 mM), 15, 33 and 54 min, respectively. The ECAR values were then determined. The data were analyzed using the Agilent Seahorse soft.

In vivo antitumor efficacy of doxorubicin and RuZ
Athymic nu/nu(nude) female mice, ages 4-5 weeks, were purchased from the Laboratory Animal Center of the Guangdong Pharmaceutical University, China. All experimental protocols were approved by the Laboratory Animal Center of the Guangdong Pharmaceutical University and used in accordance with the regulations of the Animal Ethical and Welfare Committee (AEWC). Approximately 5 × 10^6 MDA-MB-231/Adr cells were inoculated subcutaneously into the dorsal flank of the mice to establish a subcutaneous xenograft model. When the tumor volume reached approximately 80 mm^3, the volume was determined using a digital caliper and the formula: (length × $width^2$ × 1/2), the mice were randomly allocated into four groups (4 mice per group)**:** (1) PBS; (2) 3.0 mg/kg of doxorubicin (Dox); (3) 1.5 mg/kg of RuZ and (4) 3.0 mg/kg of RuZ. One hundred μL of RuZ was injected into the tumors of the mice by peritumor injection. The volume of the tumors and body weight of the mice were measured every 2 days for a period of 15 days.

In vivo pharmacokinetics and biodistribution
The MDA-MB-231/Adr tumor-bearing mice (N = 3) were intravenously injected with 3.0 mg/kg of RuZ, and 2, 6, 12, 24, 48 or 72 h after the i.v. administration of RuZ, the hearts, livers, spleens, lungs, kidneys and tumors were collected, weighed and digested using 2 mL of 68% nitric acid, followed by dilution in deionized water, and the Ru content was determined using by ICP-MS. For the pharmacokinetic experiments, the blood of the tumor-bearing mice was collected at different times (5 min, 20 min, 1 h, 2 h, 4 h, 6 h, 8 h, 12 h, 24 h) after the i.v. administration of RuZ. The blood and plasme was then collected. The plasma samples were dissolved in 2 mL of 68% nitric acid and further diluted in MQ water to measure the Ru content by ICP-MS.

Hemolysis test

Red blood cells were collected from healthy mice and incubated with various concentrations of RuZ in tubes at 37°C for 1 h. Red blood cells were incubated with either PBS or distilled water and were used as a negative (-) or postivie control (+), respectively. After incubation of the red blood cells for 3 h at room temperature, the solutions were centrifuged at 4500 rpm for 5 min at room temperature to obtain a supernatant. Subsequently, the samples were photographed and the supernatant absorbances at 541 nm was done using a by UV-visible spectrometer.

Blood biochemistry and determination of hematological parameters
The mice were intravenously injected with 3.0 mg/kg of RuZ. After 14 days, the blood was collected and was analyzed using a Auto Hematology Analyzer (Mindray, BC-2800vet) to determine blood biochemistry.

Excretion study
The mice (N = 3) were intravenously injected with 3.0 mg/kg of RuZ and the levels of RuZ in the urine and feces were determined 2, 4, 8, 12, 24, 48, 72 and 96 h) after RuZ was injected. The samples were dissolved in 2 mL of 68% nitric acid and the amount of Ru was analyzed using ICP-MS.

Acute toxicity study

Healthy mice were randomly divided into four groups (n = 3) and the mice were either PBS or RuZ (3.0, 6.0 or 12 mg/kg) was given intravenously once. The change in the body weight of the mice was measured once a daily 10 days. A loss of more than 15% of the body weight or the presence of histological tissue damage was considered to be indicative of toxicity.

Histological examination

At the end of the treatment period, all mice were euthanized using carbon dioxide. The tumors, hearts, livers, spleens, lungs, intestines and kidneys of the mice in groups 1-4 were collected and placed in 4% paraformaldehyde in a 5 mL centrifuge tube. The samples were then sent to Servicebio (Guangzhou, China) and the slices were for viewing of the morphological structures of the tissues using a light microscope (Leica DM4B).

Supporting Figures and Tables:

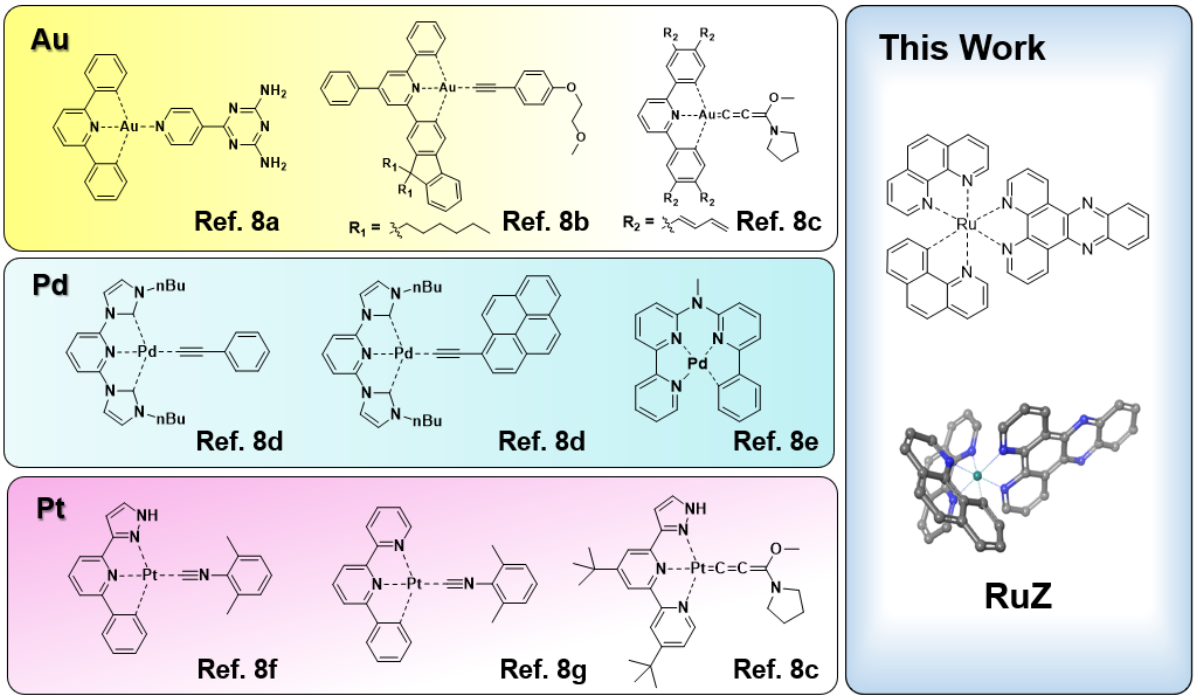

Figure S1. The structures of RuZ and the representative cyclometalated complexes that can spontaneously self-assemble into nanodrugs in water medium.[8]

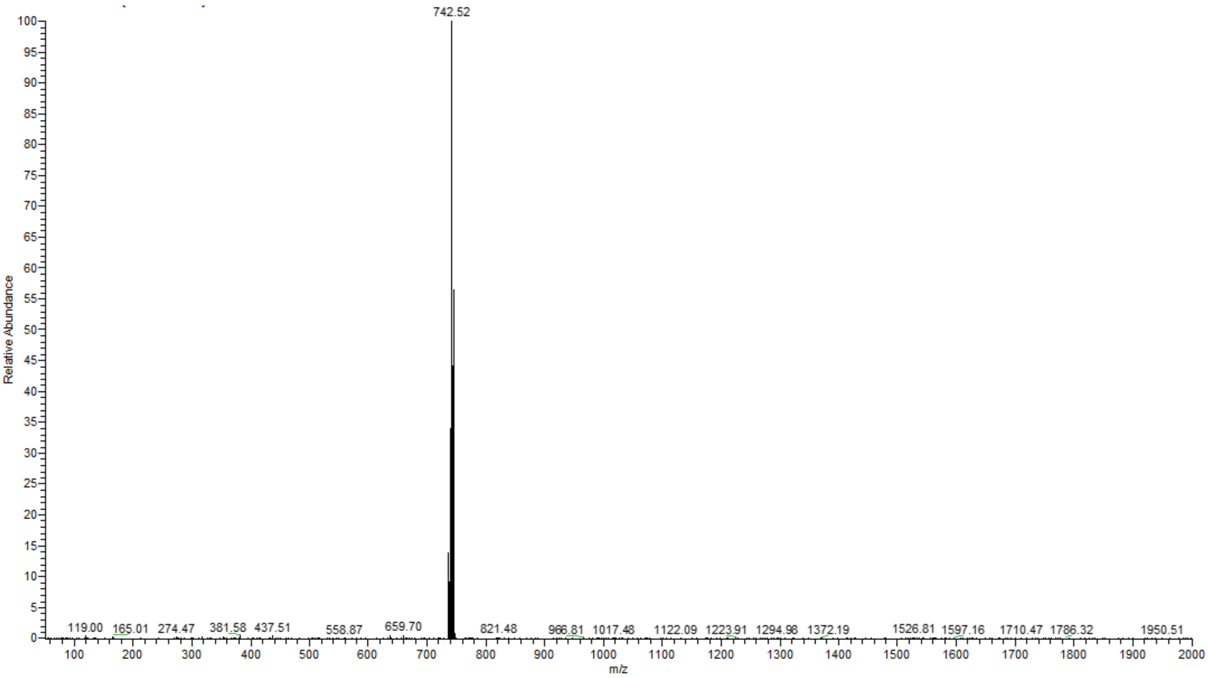

Figure S2. The ESI-MS spectrum of RuZ in methanol.

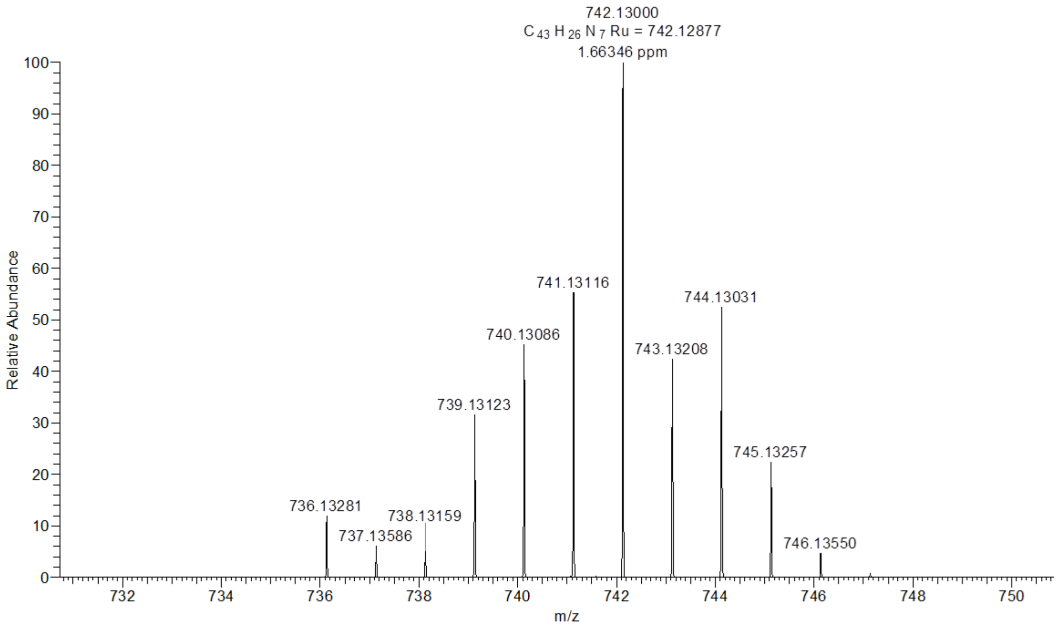

Figure S3. The HRMS spectrum of RuZ in methanol.

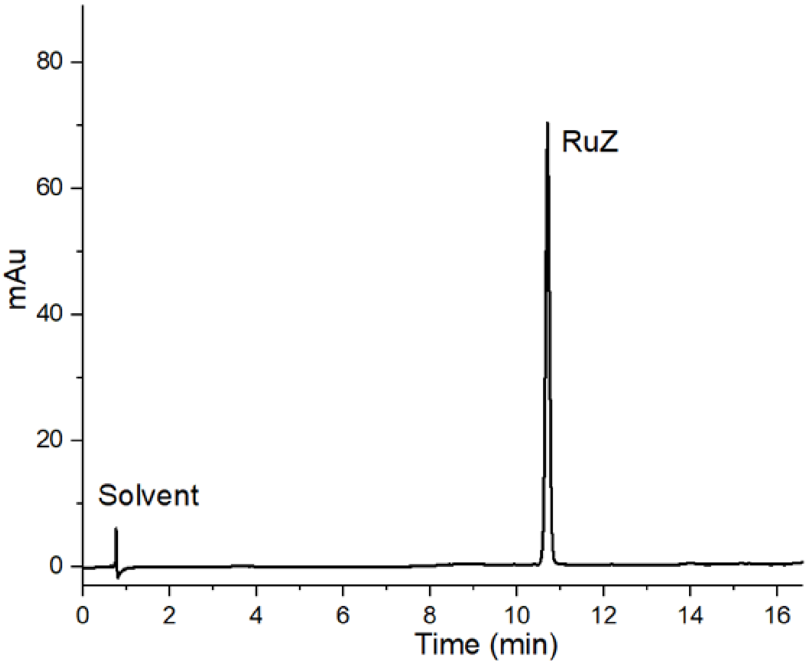

Figure S4. HPLC trace for RuZ. The elution gradient was 0-5 min and the mobile phase B: 25%; 6-10 min, B: 50%; 10-17 min, B: 100%. The mobile phases were A: water, containing 0.02% TFA-0.05% HCOOH, and B: acetonitrile containing 0.1% TFA. A flow rate of 0.5-2 mL/min was used. The purity of RuZ in this trace was > 98%.

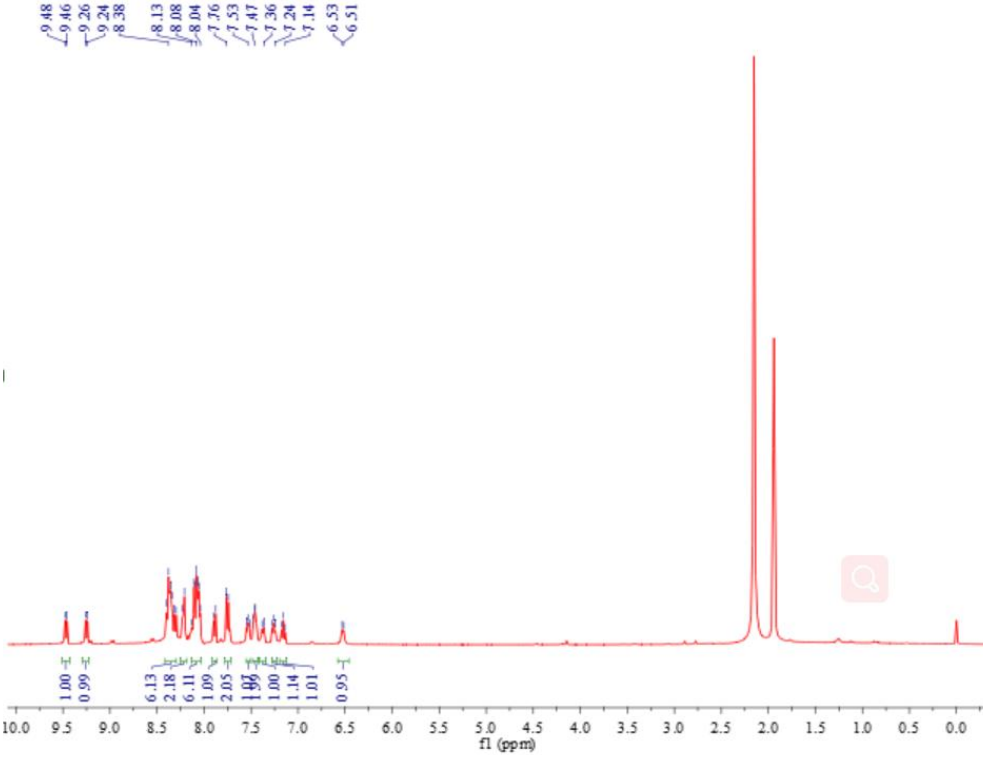

Figure S5. The ^{1}H NMR spectrum of RuZ in CD_3CN.

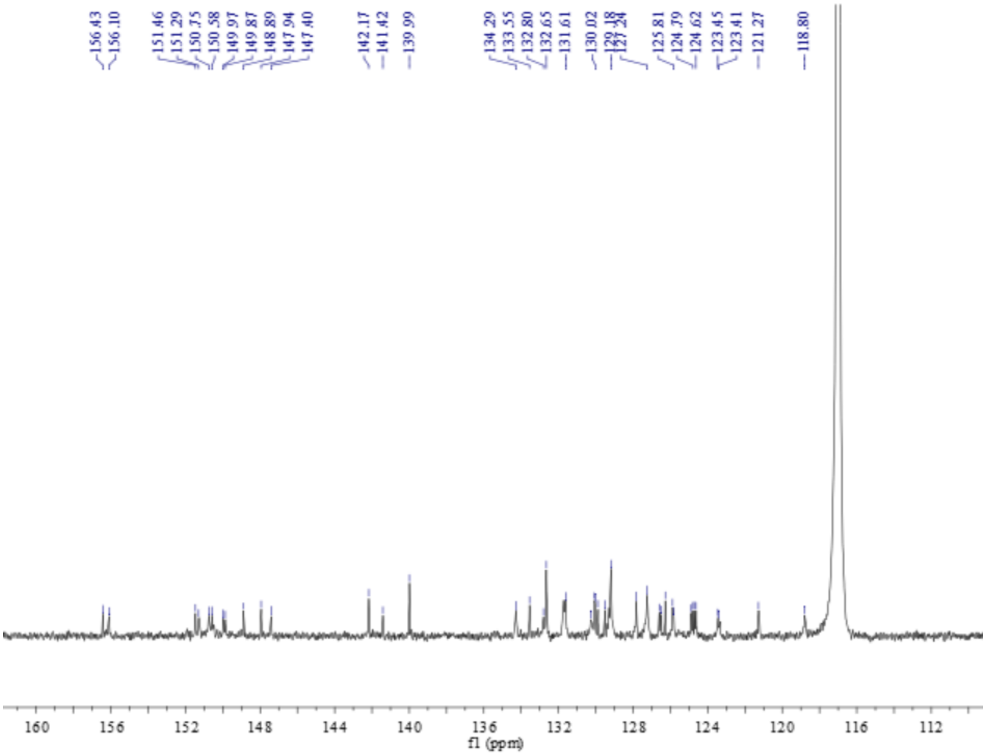

Figure S6. The ^{13}C NMR spectrum of RuZ in CD_3CN.

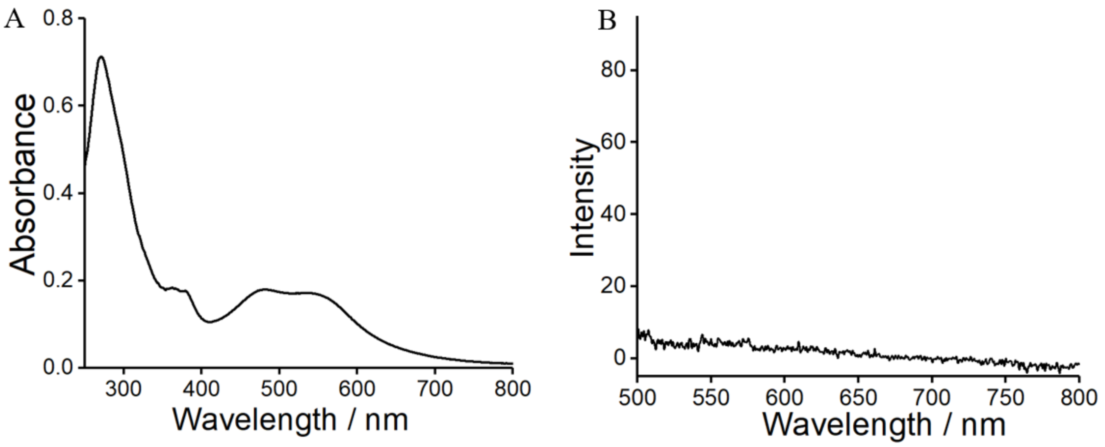

Figure S7. The absorption and emission spectrums for RuZ in PBS.

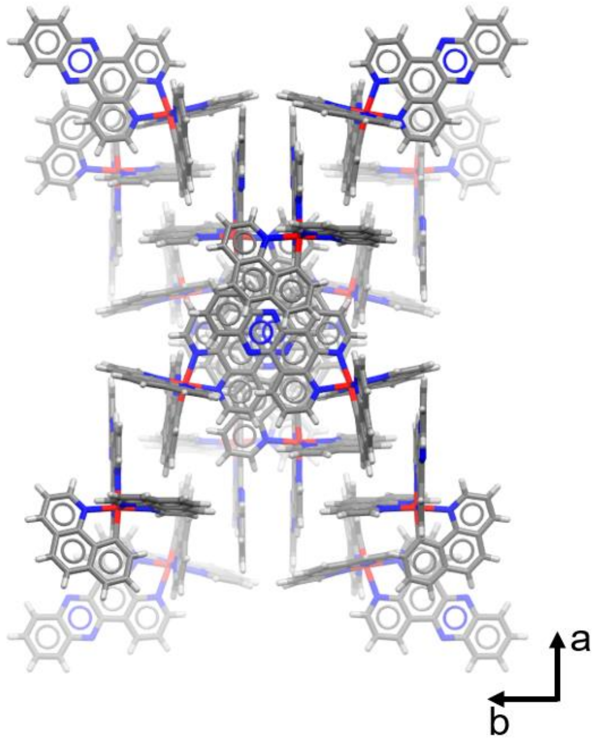

Figure S8. The stacking image of RuZ molecules in the ab plane.

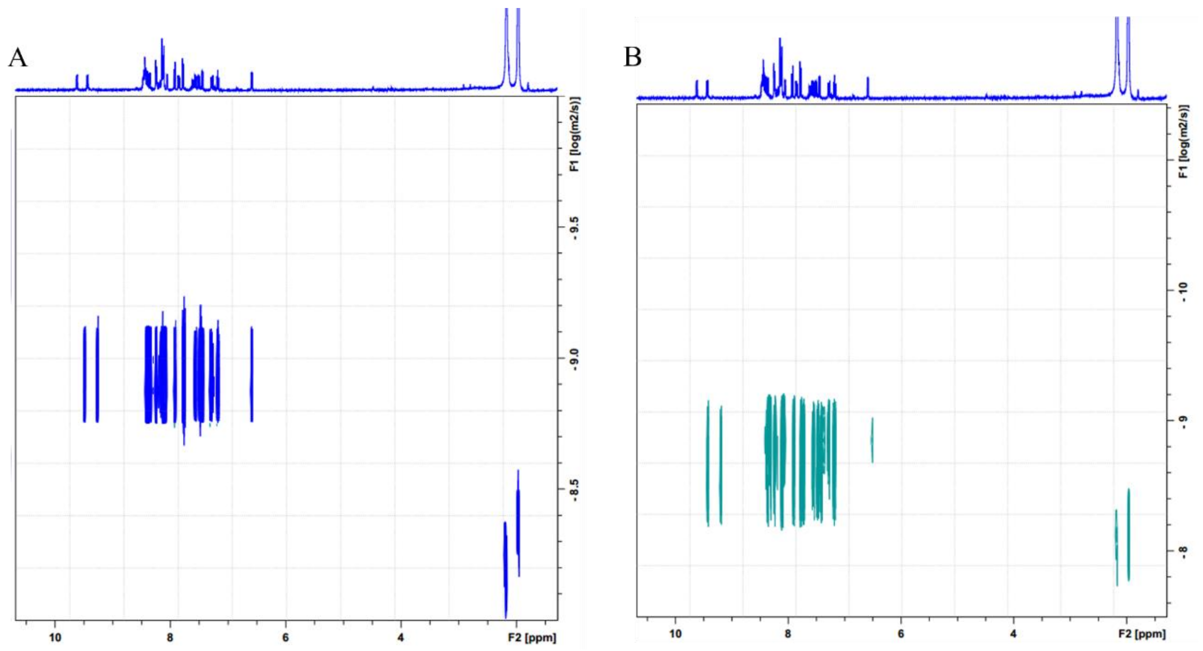

Figure S9. 2D DOSY NMR spectra (CD_3CN, room temperature, 400 MHz) of RuZ at 1 mg/0.5 mL (A) and 15 mg/0.5 mL (B).

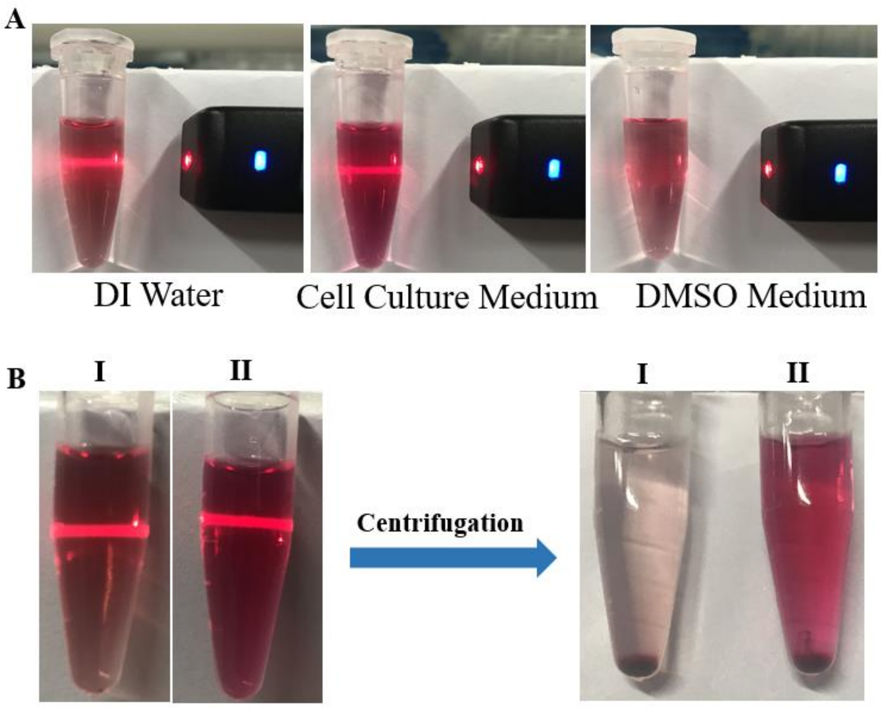

Figure S10. A) Tyndall effect of RuZ in deionized (DI) water, cell culture medium (DMEM, 10% FBS) and DMSO. B) Digital photos of RuZ in DI water and cell culture medium solutions before (left) and after (right) centrifugation. Compared with the pink color of RuZ solution, the dark red precipitate indicated the successful self-assembly of RuZ. Importantly, the colorless supernatant after centrifugation confirms the self-assembly of RuZ without obvious leakage.

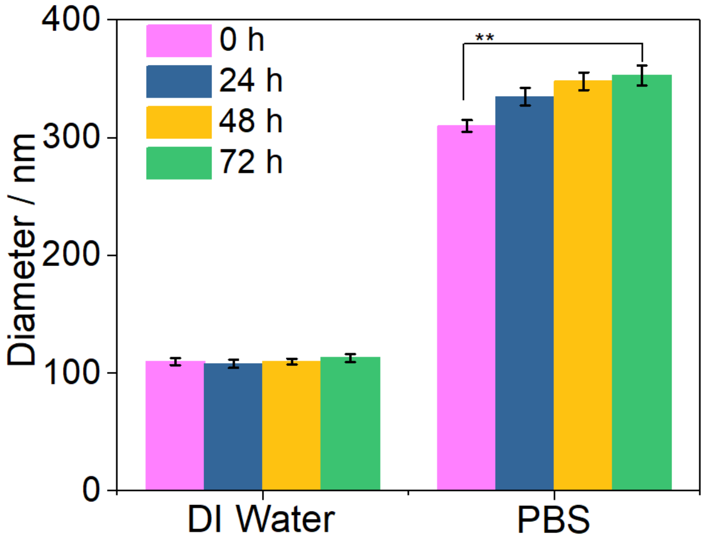

Figure S11. The mean hydrodynamic diameter of RuZ after 0, 24, 48 or 72 h of incubation in DI water and PBS.

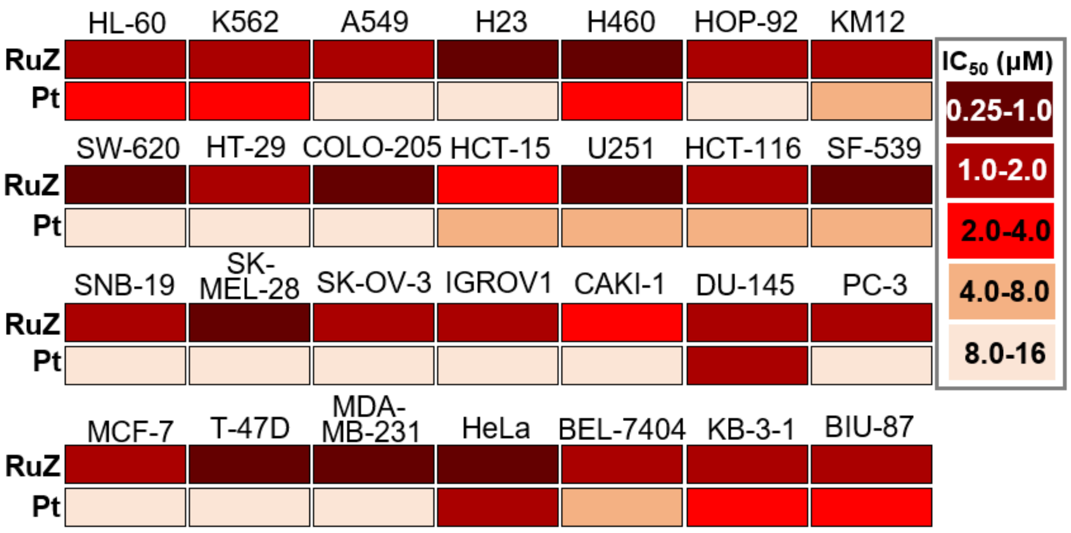

Figure S12. A) Cancer cells were incubated with RuZ or cisplatin (Pt) for 48 h and the concentration required to inhibit cell viability by 50% (IC_{50}) (n = 3, mean ± SD) was determined from three independent experiments. The five different colors represent five different IC_{50} ranges.

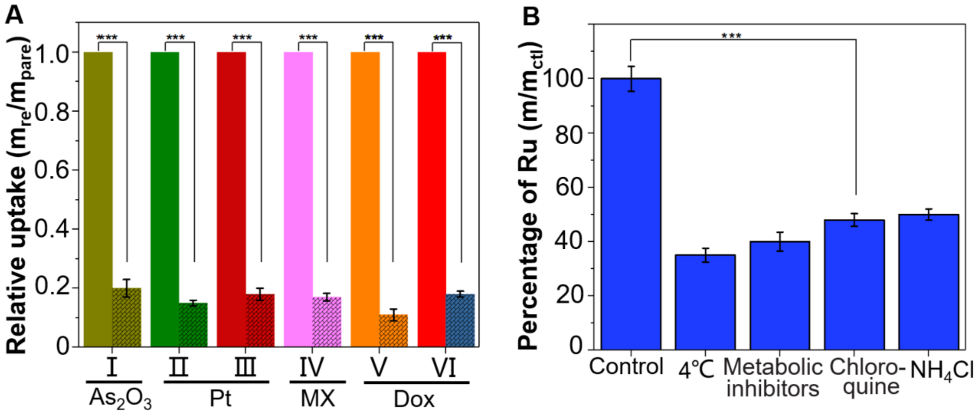

Figure S13. A) The relative uptake of drugs in resistant cells (m_{re}), compared to the uptake in parental cells (m_{pare}) after incubation 2.5 μM of As_2O_3, 2.5 μM of Pt, 1 μM of MX or 1 μM of Dox for 2 h. (n = 3, mean ± SD). B) The uptake of ruthenium in MDA-MB-231/Adr cells incubated with 2.5 μM of RuZ in the presence of either 2-deoxy-2-glucose (2-DG), chloroquine (an endocytosis inhibitor) or NH_4Cl (an endocytosis inhibitor), at 4 degrees Centigrade. *p < 0.05, ***p < 0.0001.

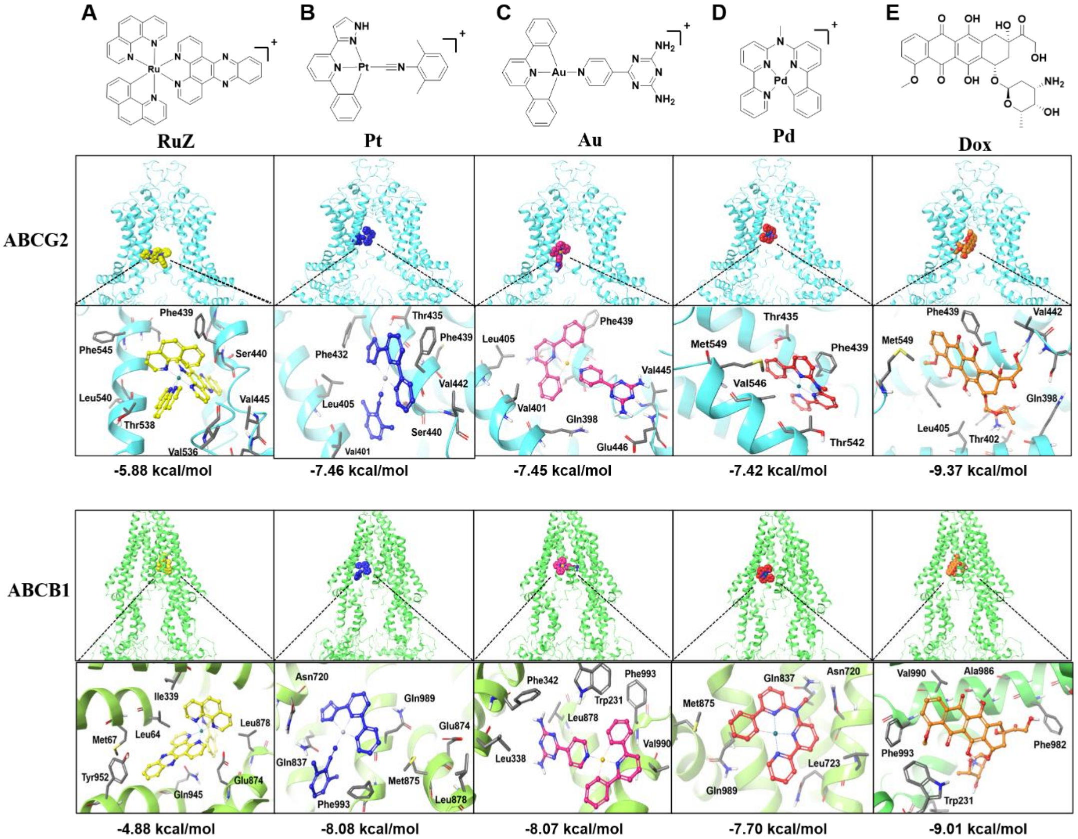

Figure S14. The overall docking positions of RuZ (A), Dox (E) and three other self-assembled anticancer cyclometalated (Pt, Au and Pd) complexes (B, C, D). All the compounds were docked at the transmembrane domain (TMD) of the ABCG2 (light blue ribbon, PDB Code: 6FFC) and ABCB1 transporter (green ribbon; PDB Code: 6FN1) under the same conditions.

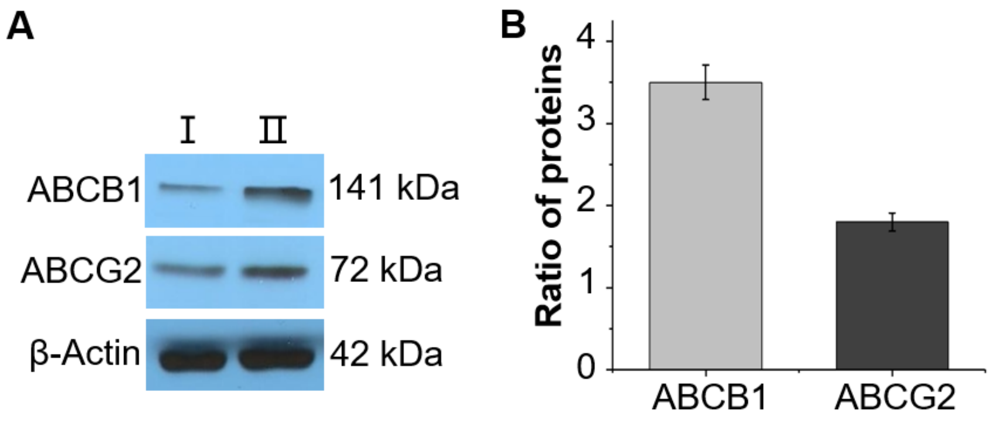

Figure S15. A) Western blot analysis of ABCB1 and ABCG2 protein expression in MDA-MB-231 (group I) and MDA-MB-231/Adr (group II) cancer cells. B) The ratio of ABCB1 or ABCG2 protein expression in MDA-MB-231 and MDA-MB-231/Adr cancer cells from A.

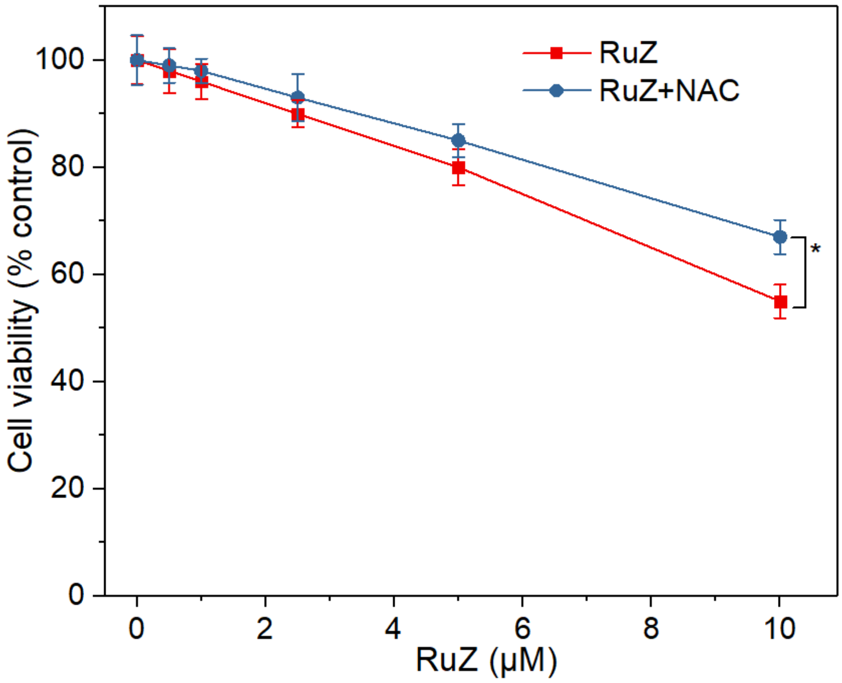

Figure S16. The effect of N-acetylcysteine (NAC) on the cytotoxicity of RuZ in MDA-MB-231/Adr cancer cells was determined following incubation with different concentrations of RuZ in the absence or presence of 5 mM of NAC for 12 h. (n = 3, mean ± SD). $^*p < 0.05$.

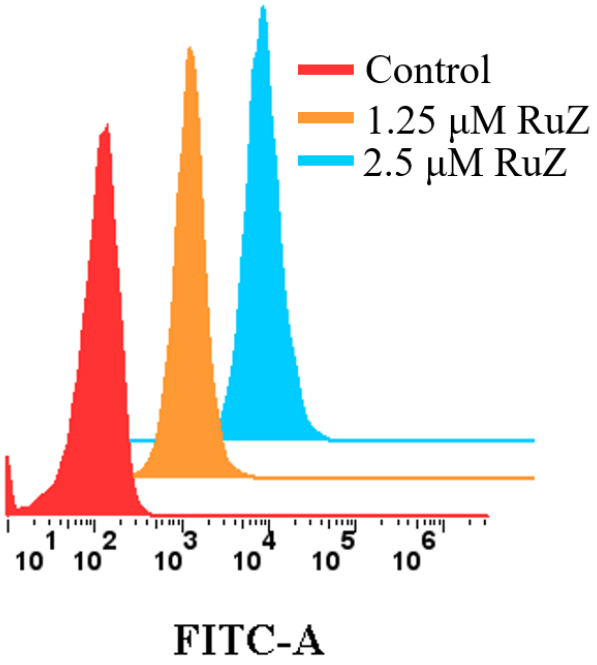

Figure S17. The effect of 1.25 or 2.5 μM of RuZ on the intracellular level of ROS in MDA-MB-231/Adr cancer cells as determined using the DCHF-DA assay and flow cytometry.

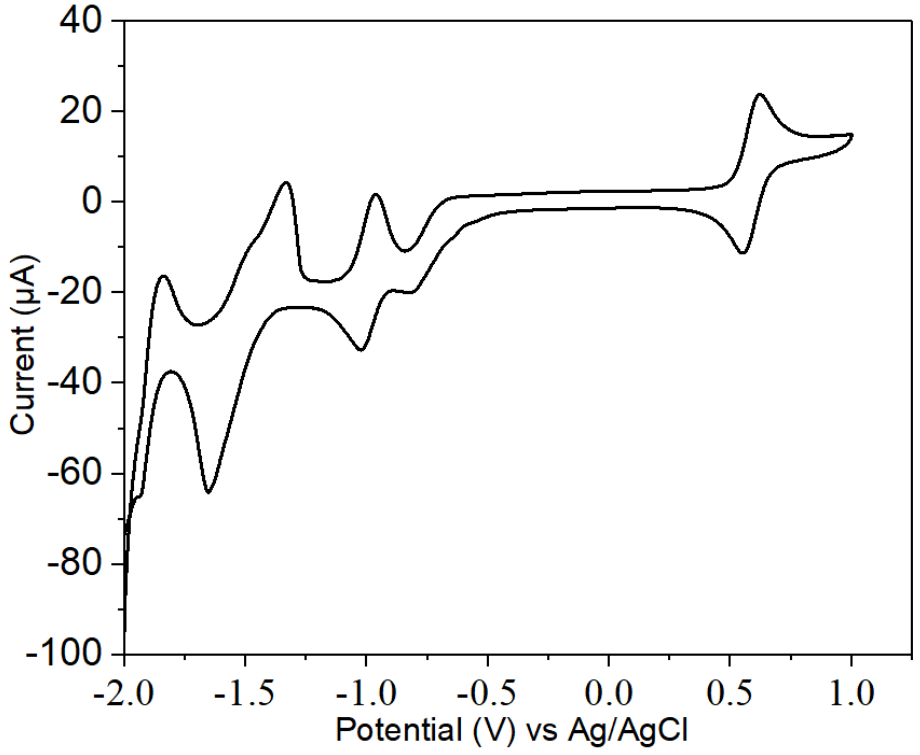

Figure S18. Cyclic Voltammogram of RuZ in MeCN (0.1 M $[Bu_4N][PF_6]$, 0.2 V/s).

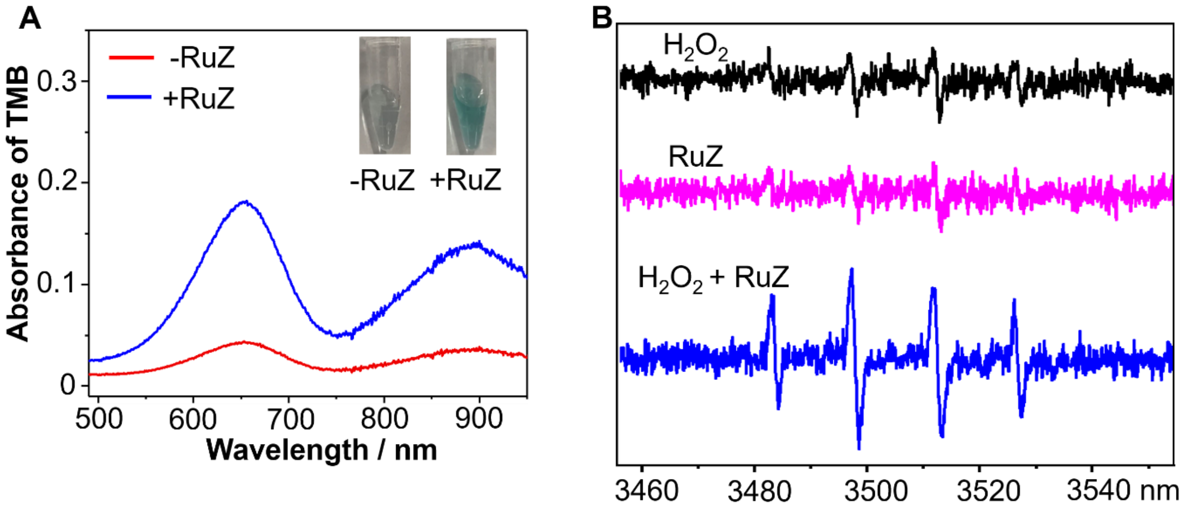

Figure S19. A) The absorption spectra of tetramethylbenzidine hydrochloride (TMB) and H_2O_2 in the presence or absence of 10 μM of RuZ for 1 h (TMB: 2.5 mg/mL, H_2O_2: 0.5 mM). Inset: photographs of the aqueous solutions. B) Electron spin resonance (ESR) spectra for the different groups incubated with 10 μL of 5,5-dimethyl-1-pyrroline N-oxide (DMPO) for 1 h (H_2O_2: 0.5 mM, RuZ: 10 μM).

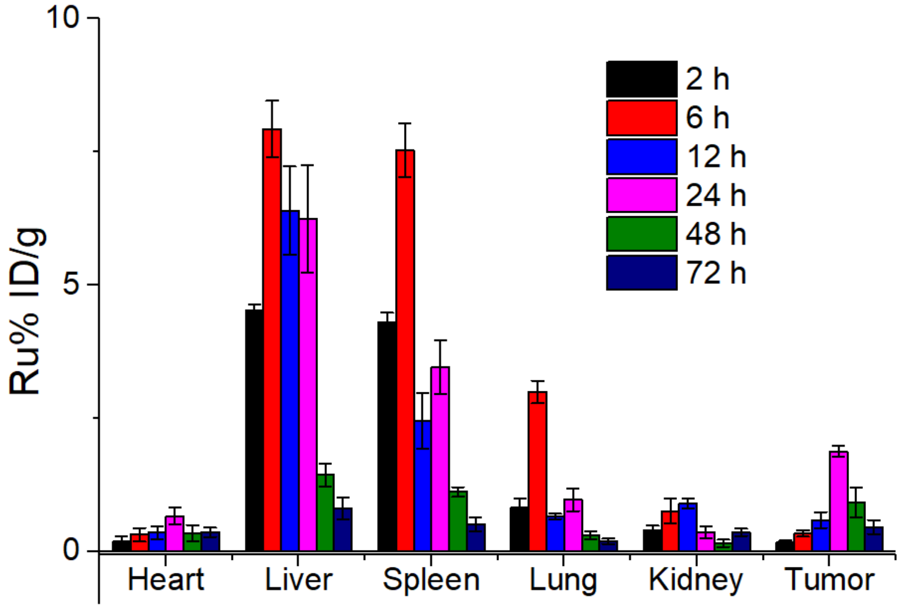

Figure S20. Ruthenium levels in the hearts, livers, spleen, lungs and kidneys and tumors following the intravenous injection of 3.0 mg/kg of RuZ in mice (n = 3), 2, 6, 8, 12, 24, 48 and 72 h after RuZ administration. The values represent the mean ± SD.

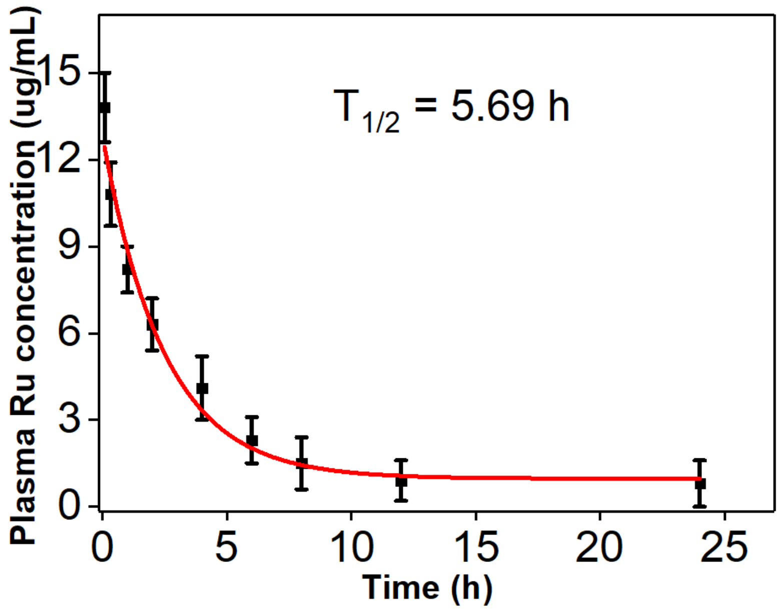

Figure S21. The levels of ruthenium in the plasma of mice (n = 3) following the i.v. administration of 3.0 mg/kg of RuZ.

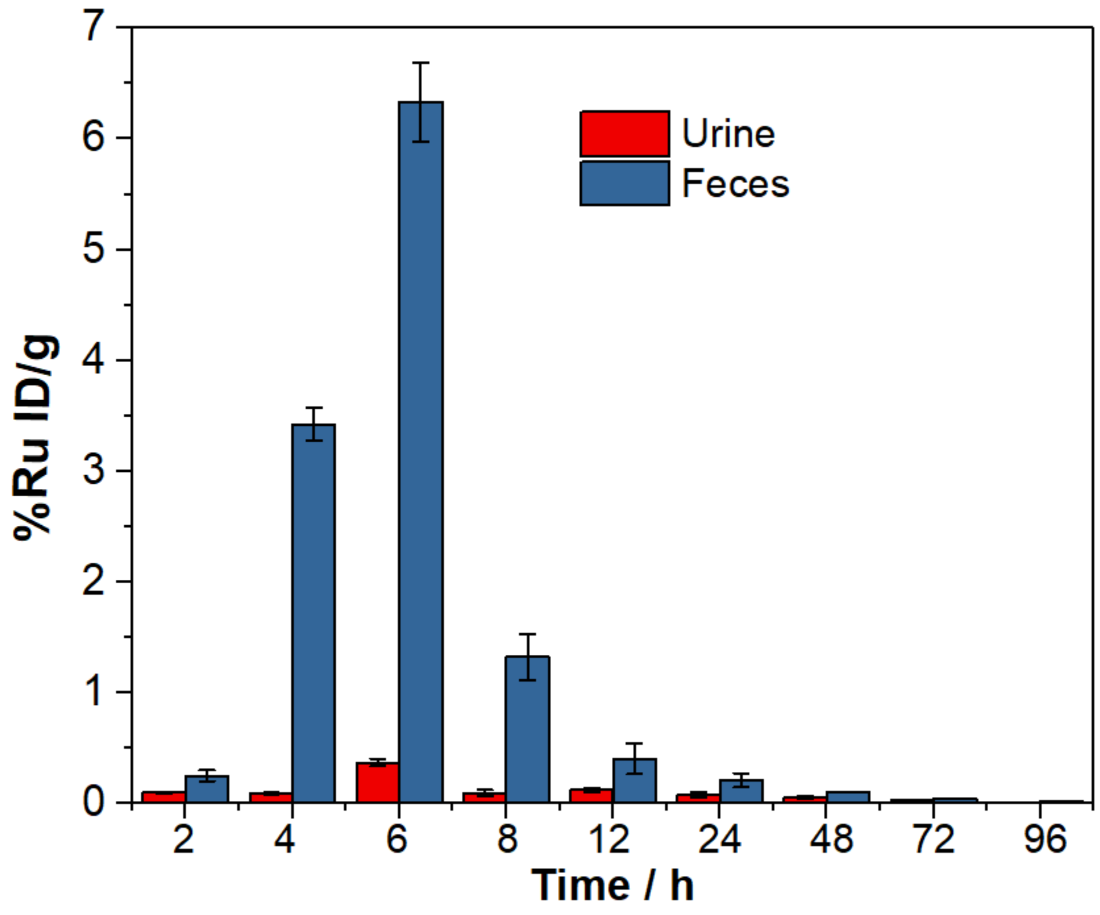

Figure S22. The levels of ruthenium in the urine (n = 3) and feces (n = 3) after the i.v. administration of 3.0 mg/kg of RuZ at different time points. The data represent the mean ± SD.

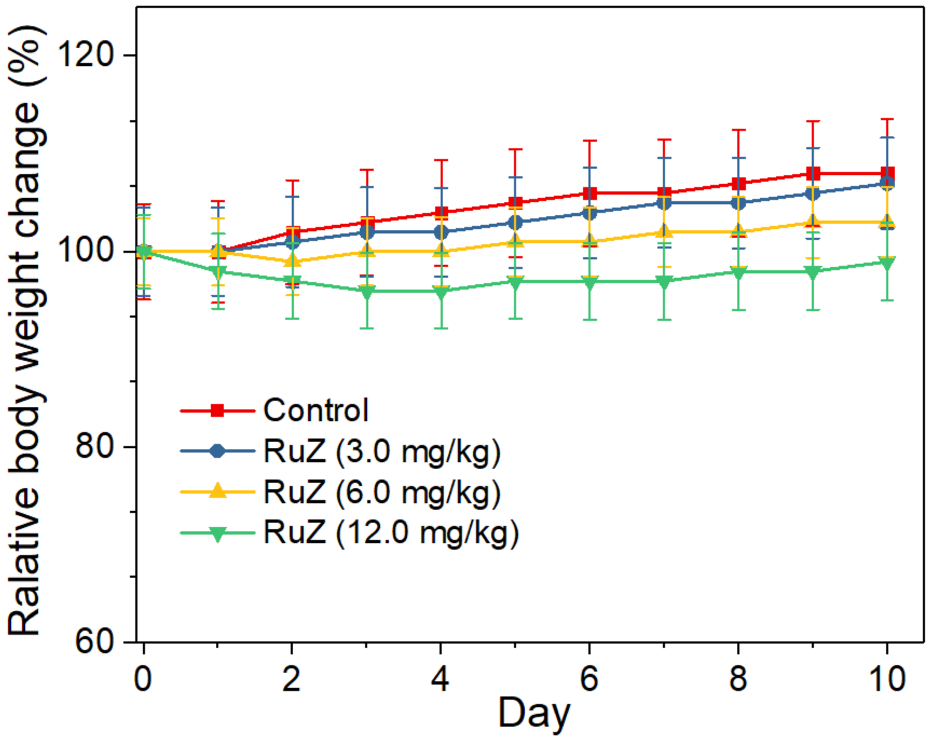

Figure S23. The effect of intravenously administered vehicle (control) or 3.0, 6.0 or 12 mg/kg of RuZ, after 10 days, on the body weight of the mice (n = 3). The data represent the mean ± SD.

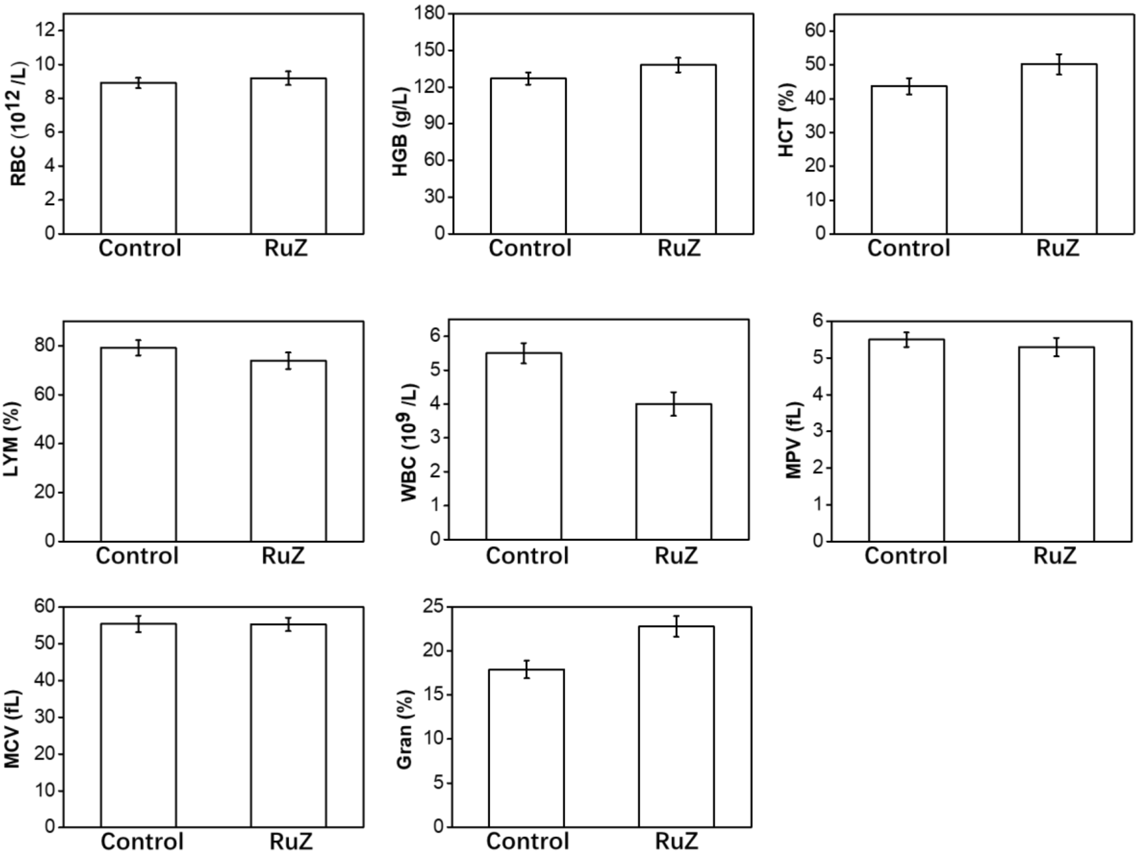

Figure S24. The effect of the i.v. administration of 3.0 mg/kg of RuZ, after 14 days, in mice (n = 3), on the number of red blood cell (RBC), hemoglobin (HGB) level, percent (%) hematocrit (HCT), the mean cellular volume (MCV) of red blood cells, the percent lymphocytes (LYM), the number of white blood cells (WBC), the mean platelet volume (MPV) and the percent (%) granulocytes. The data represent the mean ± SD.

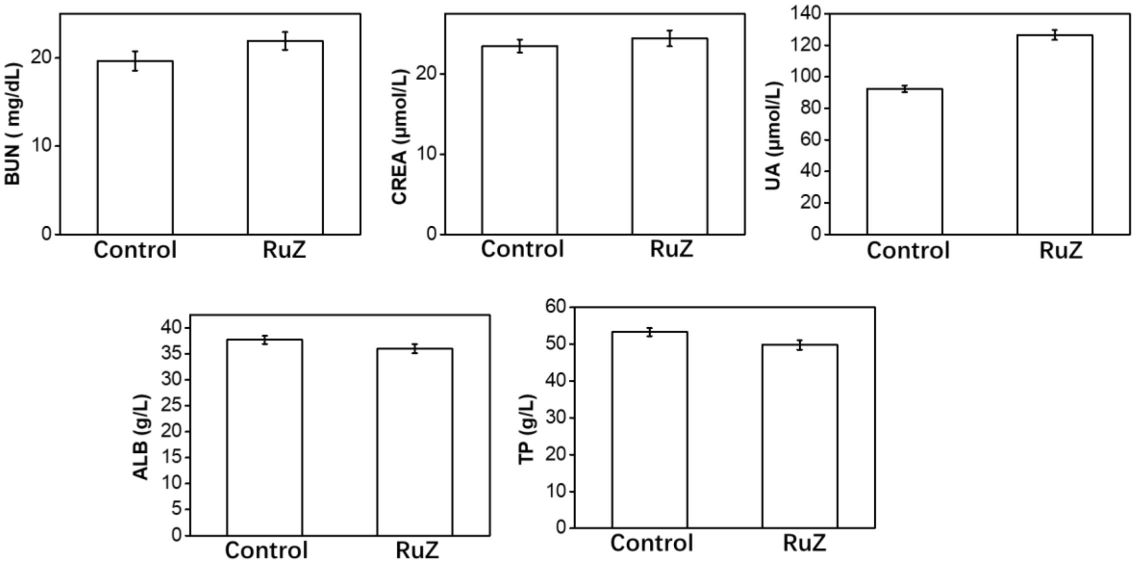

Figure S25.The effect of the intravenous administration of 3.0 mg/kg of RuZ, after 14 days, on the plasma levels of blood urea nitrogen (BUN), creatinine (CREAT), uric acid (UA), plasma albumin (ALB) and total protein (TP), in mice (n = 3). The values represent the mean ± SD.

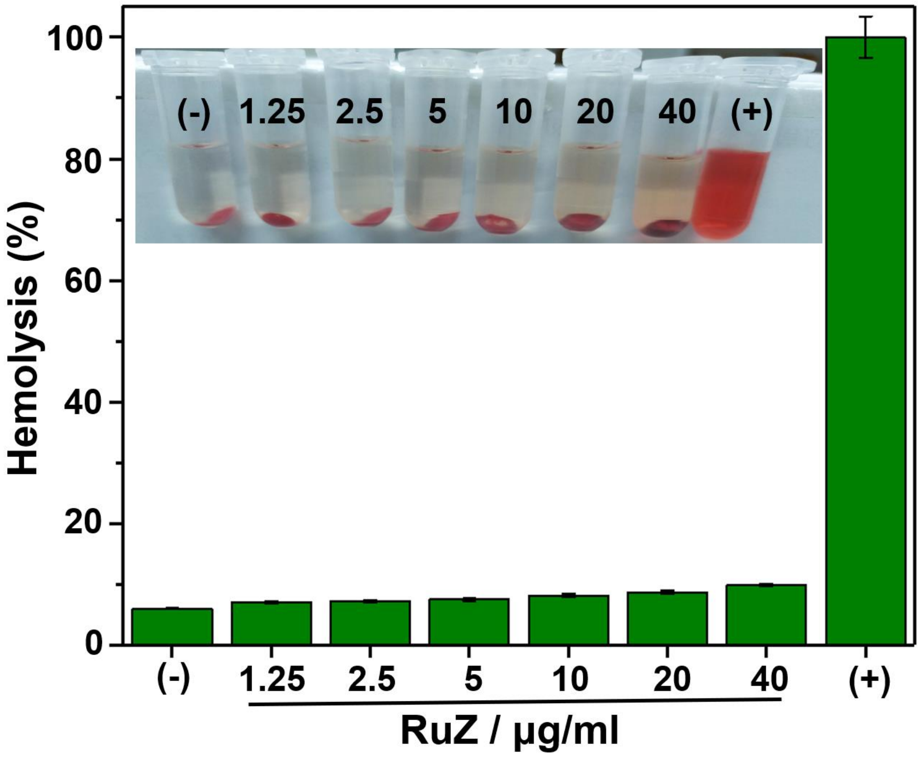

Figure S26. The determination of the magnitude of red blood cell hemolysis following incubation with the negative (-) and positive (+) controls, PBS (n = 3) and vehicle (n = 3), respectively, and RuZ (n = 3) for1 h. The values represent the mean ± SD.

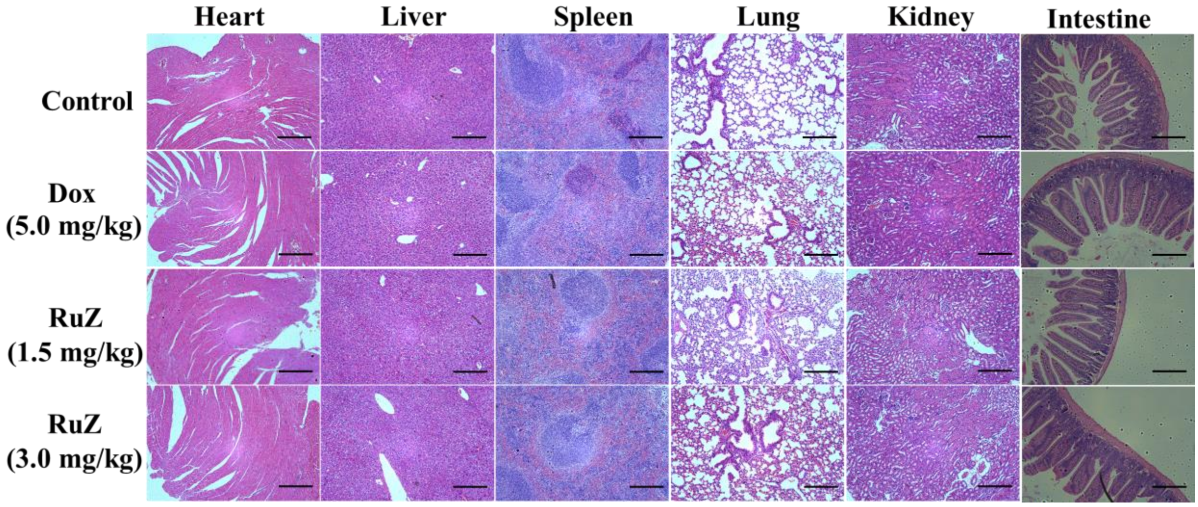

Figure S27. Representative images of hematoxylin and eosin staining of tissue from the hearts, livers, spleens, lungs, kidneys and intestines of mice that were given with vehicle (Control), 5.0 mg/kg of Dox, 1.5 or 3.0 mg/kg of RuZ, peritumorally, for 14 days. The mice were sacrificed by using carbon dioxide after the peritumoral injection on Day 14. Scale bar = 250 μm.

Table S1 The effect of RuZ and cisplatin on the viability of normal liver LO2 and MCF10A cells.[a]

Treatments	IC_{50} ± SD (μM)	
	LO2	MCF10A
**RuZ**	7.46±0.41	8.52±0.72
**Cisplatin**	6.52±0.56	7.72±0.83

[a] The cells were incubated with various concentrations of either RuZ or cisplatin for 48 h. Each value represents the mean ± SD of three independent experiments.

Table S2 The cytotoxicity of mitoxantrone, doxorubicin and cisplatin in seven parental and drug-resistant cancer cell lines.

**Drugs**	**Cell lines / IC_{50}±SD (μM)** [a]		**RF**[b]	**Drugs**	**Cell lines / IC_{50}±SD (μM)**		**RF**
	Parental cells	Resistant cells			Parental cells	Resistant cells	
	**H460**	**H460/MX20**			**KB-3-1**	**KB/ATO**	
**MX**	0.10±0.013	5.52±0.62	55	**As_2O_3**	2.01±0.37	18.04±0.83	9
	**KB-3-1**	**KB-C-2**			**BEL-7404**	**BEL-7404/CP20**	
**Dox**	0.16±0.018	5.62±0.44	35	**Pt**	5.2±0.48	50±2.27	10
	**SW620**	**SW620/AD300**			**BIU-87**	**BIU-87/DDP**	
**Dox**	0.23±0.028	6.81±0.57	30	**Pt**	3.35±0.38	28.48±1.37	8.5
	**MDA-MB-231**	**MDA-MB-231/Adr**					
**Dox**	0.35±0.12	8.5±0.43	24				

[a]: The cells were incubated with the appropriate drug for 48 h. Each value represents the mean ± SD of three independent experiments. MX = mitoxantrone, Dox = doxorubicin and Pt = cisplatin. [b]: RF was calculated by dividing the IC_{50} value for the drug-resistant cancer cell by the IC_{50} value for parental cancer cell.

Table S3 Crystal data and structure refinement for RuZ.

CCDC number	2031773
Empirical formula	$C_{43}H_{26}F_6N_7PRu$
Formula weight	886.75
Temperature/K	150
Crystal system	monoclinic
Space group	C2/c
a/Å	23.4435(8)
b/Å	33.7296(11)
c/Å	21.9842(7)
α/°	90
β/°	106.112(3)
γ/°	90
Volume/Å^3	16701.0(10)
Z	16
ρ_{calc}g/cm^3	1.411
μ/mm^{-1}	3.972
F (000)	7136.0
Crystal size/mm^3	0.18 × 0.12 × 0.11
Radiation	CuKα (λ = 1.54184)
2Θ range for data collection/°	4.718 to 132.012
Index ranges	-27 ≤ h ≤ 27, -39 ≤ k ≤ 39, -26 ≤ l ≤ 24
Reflections collected	64835
Independent reflections	14450 [R_{int} = 0.0593, R_{sigma} = 0.0475]
Data/restraints/parameters	14450/190/1074
Goodness-of-fit on F^2	1.119
Final R indexes [I>=2σ (I)]	R_1 = 0.1178, wR_2 = 0.2308
Final R indexes [all data]	R_1 = 0.1410, wR_2 = 0.2418
Largest diff. peak/hole / e Å^{-3}	1.85/-1.73

Table S4 Bond Lengths for RuZ.

Atom	Atom	Length/Å	Atom	Atom	Length/Å
Ru1	N1	2.104(8)	N10	C55	1.355(13)
Ru1	N2	2.110(9)	N10	C61	1.390(15)
Ru1	N5	2.085(9)	N11	C54	1.359(13)
Ru1	N6	2.078(9)	N11	C56	1.355(15)
Ru1	N7	2.058(9)	N12	C62	1.341(13)
Ru1	C29	2.042(10)	N12	C74	1.391(13)
N1	C1	1.343(13)	N13	C75	1.377(13)
N1	C5	1.365(12)	N13	C86	1.399(12)
N2	C6	1.403(13)	N14	C84	1.357(13)
N2	C10	1.321(14)	N14	C85	1.396(12)
N3	C12	1.364(13)	C44	C45	1.406(15)

N3	C18	1.375(14)	C45	C46	1.399(15)
N4	C11	1.324(13)	C46	C47	1.426(14)
N4	C13	1.379(14)	C47	C48	1.401(14)
N5	C19	1.336(14)	C47	C55	1.477(14)
N5	C31	1.401(13)	C48	C49	1.443(13)
N6	C32	1.344(14)	C49	C50	1.418(14)
N6	C43	1.411(13)	C50	C51	1.404(15)
N7	C41	1.355(13)	C50	C54	1.478(15)
N7	C42	1.393(12)	C51	C52	1.386(16)
C1	C2	1.415(14)	C52	C53	1.386(16)
C2	C3	1.397(14)	C54	C55	1.419(14)
C3	C4	1.391(15)	C56	C57	1.465(17)
C4	C5	1.425(13)	C56	C61	1.433(17)
C4	C11	1.491(14)	C57	C58	1.382(19)
C5	C6	1.470(14)	C58	C59	1.43(2)
C6	C7	1.420(14)	C59	C60	1.431(19)
C7	C8	1.407(15)	C60	C61	1.414(17)
C7	C12	1.491(15)	C62	C63	1.409(16)
C8	C9	1.385(16)	C63	C64	1.370(18)
C9	C10	1.425(16)	C64	C65	1.396(17)
C11	C12	1.450(16)	C65	C66	1.436(17)
C13	C14	1.455(15)	C65	C74	1.437(14)
C13	C18	1.467(16)	C66	C67	1.360(17)
C14	C15	1.373(16)	C67	C68	1.463(15)
C15	C16	1.415(19)	C68	C69	1.422(16)
C16	C17	1.385(17)	C68	C73	1.445(15)
C17	C18	1.392(15)	C69	C70	1.374(15)
C19	C20	1.429(16)	C70	C71	1.452(16)
C20	C21	1.384(17)	C71	C72	1.391(15)
C21	C22	1.404(17)	C72	C73	1.435(14)
C22	C23	1.426(16)	C73	C74	1.447(15)
C22	C31	1.432(14)	C75	C76	1.394(14)
C23	C24	1.359(15)	C76	C77	1.376(14)
C24	C25	1.455(14)	C77	C78	1.418(14)
C25	C26	1.404(15)	C78	C79	1.440(14)
C25	C30	1.438(14)	C78	C86	1.400(13)
C26	C27	1.384(15)	C79	C80	1.365(15)
C27	C28	1.387(14)	C80	C81	1.435(14)
C28	C29	1.378(13)	C81	C82	1.422(14)
C29	C30	1.392(13)	C81	C85	1.411(13)
C30	C31	1.417(14)	C82	C83	1.364(14)
C32	C33	1.433(15)	C83	C84	1.401(14)
C33	C34	1.365(17)	C85	C86	1.451(13)
C34	C35	1.415(16)	P1	F1	1.518(11)
C35	C36	1.444(16)	P1	F2	1.588(11)
C35	C43	1.422(14)	P1	F3	1.591(11)

C36	C37	1.346(18)	P1	F4	1.525(11)
C37	C38	1.462(16)	P1	F5	1.622(11)
C38	C39	1.405(16)	P1	F6	1.557(11)
C38	C42	1.434(14)	P2	F7	1.441(15)
C39	C40	1.355(16)	P2	F8	1.653(18)
C40	C41	1.402(14)	P2	F9	1.81(3)
C42	C43	1.395(15)	P2	F10	1.49(3)
Ru2	N8	2.116(9)	P2	F11	1.70(3)
Ru2	N9	2.074(8)	P2	F12	1.83(3)
Ru2	N12	2.100(9)	P3	F13	1.585(16)
Ru2	N13	2.040(8)	P3	F14	1.675(17)
Ru2	N14	2.075(8)	P3	F15	1.602(16)
Ru2	C72	2.036(11)	P3	F16	1.771(17)
N8	C44	1.321(13)	P3	F17	1.561(17)
N8	C48	1.389(12)	P3	F18	1.653(18)
N9	C49	1.386(13)	F16	F18[1]	1.67(4)
N9	C53	1.353(13)	F18	F16[1]	1.67(4)

Author Contributions

L. Zeng, H. Chao, Z-S Chen, and Y. Pan conceived the idea, proposed the strategy. L. Zeng performed the experiments and wrote the manuscript. L. Zeng, Y. Pan and Z-S Chen prepared the drug-resistant cell systems. J. Li synthesized RuZ and conducted the cell experiments and was involved in writing the manuscript. J. Li and J. Wang helped with the animal experiments. C-Y. Cai and E. Xing analyzed the molecular docking data. C Ashby and Y. Pan were involved in writing the manuscript. Z. Wang and Z. Li helped analyze the single crystal data. H. Chen and S. Fang helped analyze the proteomic profiling data and X. Liao helped draw the scheme and TOC picture.

References

[1] O. V. Dolomanov, L. J. Bourhis, R. J. Gildea, J. A. K. Howard and H. Puschmann, *J. Appl. Cryst.* **2009**, *42*, 339-341.
[2] G. Sheldrick, *Acta Cryst.* **2015**, *C71*, 3-8.
[3] Y.-K. Zhang, G.-N. Zhang, Y.-J. Wang, B. A. Patel, T. T. Talele, D.-H. Yang and Z.-S. Chen, *Sci. Rep.* **2016**, *6*, 25694.
[4] Y.-F. Fan, W. Zhang, L. Zeng, Z.-N. Lei, C.-Y. Cai, P. Gupta, D.-H. Yang, Q. Cui, Z.-D. Qin, Z.-S. Chen and L. D. Trombetta, *Cancer Lett.* **2018**, *421*, 186-198.
[5] L. He, M. F. Zhang, Z. Y. Pan, K. N. Wang, Z. J. Zhao, Y. Li and Z. W. Mao, *Chem. Commun.* **2019**, *55*, 10472-10475.
[6] H. Huang, P. Zhang, B. Yu, Y. Chen, J. Wang, L. Ji and H. Chao, *J. Med. Chem.* **2014**, *57*, 8971-8983.
[7] J. Liu, Y. Chen, G. Li, P. Zhang, C. Jin, L. Zeng, L. Ji and H. Chao, *Biomaterials* **2015**, *56*, 140-153.
[8] a) J. J. Zhang, W. Lu, R. W. Y. Sun and C. M. Che, *Angew. Chem.* **2012**, *124*, 4966-4970; b) F. Wang, M. Lan, W.-P. To, K. Li, C.-N. Lok, P. Wang and C.-M. Che, *Chem. Commun.* **2016**, *52*, 13273-13276; c) X.-S. Xiao, W.-L. Kwong, X. Guan, C. Yang, W. Lu and C.-M. Che, *Chem– Eur. J.* **2013**, *19*, 9457-9462; d) F. F. Hung, S. X. Wu, W. P. To, W. L. Kwong, X. Guan, W. Lu, K. H. Low and C. M. Che, *ChemistryAsian J.* **2017**, *12*, 145-158; e) X. Q. Zhou, M. Xiao, V. Ramu, J. Hilgendorf, X. Li, P. Papadopoulou, M. A. Siegler, A. Kros, W. Sun and S. Bonnet, *J. Am. Chem. Soc.* **2020**, *142*, 10383-10399; f) J. L. L. Tsai, T. T. Zou, J. Liu, T. F. Chen, A. O. Y. Chan, C. Yang, C. N. Lok and C. M. Che, *Chem. Sci.* **2015**, *6*, 3823-3830; g) C. N. Lok, T. T. Zou, J. J. Zhang, I. W. S. Lin and C. M. Che, *Adv. Mater.* **2014**, *26*, 5550-5557.
