# Supplementary material for: Cycloruthenated Self‐Assembly with Metabolic Inhibition to Efficiently Overcome Multidrug Resistance in Cancers
Source: Adv Mater. 2021 Oct 24;34(1):2100245. doi: 10.1002/adma.202100245 (PMC11468970; doi:10.1002/adma.202100245)

checkCIF/PLATON report

You have not supplied any structure factors. As a result the full set of tests cannot be run.

THIS REPORT IS FOR GUIDANCE ONLY. IF USED AS PART OF A REVIEW PROCEDURE FOR PUBLICATION, IT SHOULD NOT REPLACE THE EXPERTISE OF AN EXPERIENCED CRYSTALLOGRAPHIC REFEREE.

No syntax errors found. CIF dictionary Interpreting this report

Datablock: 2

```
Bond precision:  C-C = 0.0173 A          Wavelength=1.54184

Cell:           a=23.4435(8)    b=33.7296(11)      c=21.9842(7)
                alpha=90        beta=106.112(3)    gamma=90
Temperature:    150 K
```

	Calculated	Reported
Volume	16701.0(10)	16701.0(10)
Space group	C 2/c	C 1 2/c 1
Hall group	-C 2yc	-C 2yc
Moiety formula	C43 H26 N7 Ru, F6 P [+ solvent]	C43 H26 N7 Ru, F6 P
Sum formula	C43 H26 F6 N7 P Ru [+ solvent]	C43 H26 F6 N7 P Ru
Mr	886.75	886.75
Dx,g cm-3	1.411	1.411
Z	16	16
Mu (mm-1)	3.972	3.972
F000	7136.0	7136.0
F000’	7163.57	
h,k,lmax	27,39,26	27,39,26
Nref	14568	14450
Tmin,Tmax	0.593,0.646	0.441,0.751
Tmin’	0.466	

```
Correction method= # Reported T Limits: Tmin=0.441 Tmax=0.751
AbsCorr = MULTI-SCAN

Data completeness= 0.992          Theta(max)= 66.006

R(reflections)= 0.1178( 10721)    wR2(reflections)= 0.2418( 14450)

S = 1.119                  Npar= 1074
```

The following ALERTS were generated. Each ALERT has the format
test-name_ALERT_alert-type_alert-level.
Click on the hyperlinks for more details of the test.

Alert level C

```
PLAT082_ALERT_2_C High R1 Value .................................        0.12 Report
PLAT234_ALERT_4_C Large Hirshfeld Difference N5       --C19      .       0.16 Ang.
PLAT234_ALERT_4_C Large Hirshfeld Difference C11      --C12      .       0.16 Ang.
PLAT234_ALERT_4_C Large Hirshfeld Difference C29      --C30      .       0.17 Ang.
PLAT234_ALERT_4_C Large Hirshfeld Difference C33      --C34      .       0.18 Ang.
PLAT234_ALERT_4_C Large Hirshfeld Difference C51      --C52      .       0.18 Ang.
PLAT234_ALERT_4_C Large Hirshfeld Difference C71      --C72      .       0.19 Ang.
PLAT234_ALERT_4_C Large Hirshfeld Difference P1       --F2       .       0.18 Ang.
PLAT234_ALERT_4_C Large Hirshfeld Difference P1       --F6       .       0.18 Ang.
PLAT241_ALERT_2_C High   'MainMol' Ueq as Compared to Neighbors of        C63 Check
PLAT260_ALERT_2_C Large Average Ueq of Residue Including        P1      0.184 Check
PLAT260_ALERT_2_C Large Average Ueq of Residue Including        P2      0.281 Check
PLAT342_ALERT_3_C Low Bond Precision on  C-C Bonds ...............    0.01728 Ang.
```

Alert level G

```
PLAT002_ALERT_2_G Number of Distance or Angle Restraints on AtSite         14 Note
PLAT003_ALERT_2_G Number of Uiso or Uij Restrained non-H Atoms ...         21 Report
PLAT083_ALERT_2_G SHELXL Second Parameter in WGHT  Unusually Large     323.00 Why ?
PLAT172_ALERT_4_G The CIF-Embedded .res File Contains DFIX Records         24 Report
PLAT174_ALERT_4_G The CIF-Embedded .res File Contains FLAT Records          2 Report
PLAT178_ALERT_4_G The CIF-Embedded .res File Contains SIMU Records          3 Report
PLAT186_ALERT_4_G The CIF-Embedded .res File Contains ISOR Records          3 Report
PLAT244_ALERT_4_G Low    'Solvent' Ueq as Compared to Neighbors of         P1 Check
PLAT300_ALERT_4_G Atom Site Occupancy of P2         Constrained at        0.5 Check
PLAT300_ALERT_4_G Atom Site Occupancy of F7         Constrained at        0.5 Check
PLAT300_ALERT_4_G Atom Site Occupancy of F8         Constrained at        0.5 Check
PLAT300_ALERT_4_G Atom Site Occupancy of F9         Constrained at        0.5 Check
PLAT300_ALERT_4_G Atom Site Occupancy of F10        Constrained at        0.5 Check
PLAT300_ALERT_4_G Atom Site Occupancy of F11        Constrained at        0.5 Check
PLAT300_ALERT_4_G Atom Site Occupancy of F12        Constrained at        0.5 Check
PLAT300_ALERT_4_G Atom Site Occupancy of P3         Constrained at        0.5 Check
PLAT300_ALERT_4_G Atom Site Occupancy of F13        Constrained at        0.5 Check
PLAT300_ALERT_4_G Atom Site Occupancy of F14        Constrained at        0.5 Check
PLAT300_ALERT_4_G Atom Site Occupancy of F15        Constrained at        0.5 Check
PLAT300_ALERT_4_G Atom Site Occupancy of F16        Constrained at        0.5 Check
PLAT300_ALERT_4_G Atom Site Occupancy of F17        Constrained at        0.5 Check
PLAT300_ALERT_4_G Atom Site Occupancy of F18        Constrained at        0.5 Check
PLAT302_ALERT_4_G Anion/Solvent/Minor-Residue Disorder (Resd  4  )       100% Note
PLAT302_ALERT_4_G Anion/Solvent/Minor-Residue Disorder (Resd  5  )       100% Note
PLAT304_ALERT_4_G Non-Integer Number of Atoms in ..... (Resd  4  )       3.50 Check
PLAT304_ALERT_4_G Non-Integer Number of Atoms in ..... (Resd  5  )       3.50 Check
PLAT333_ALERT_2_G Large Aver C6-Ring C-C Dist C4       -C11      .       1.46 Ang.
PLAT333_ALERT_2_G Large Aver C6-Ring C-C Dist C22      -C31      .       1.42 Ang.
PLAT333_ALERT_2_G Large Aver C6-Ring C-C Dist C35      -C43      .       1.42 Ang.
PLAT333_ALERT_2_G Large Aver C6-Ring C-C Dist C47      -C55      .       1.44 Ang.
PLAT333_ALERT_2_G Large Aver C6-Ring C-C Dist C56      -C61      .       1.43 Ang.
PLAT333_ALERT_2_G Large Aver C6-Ring C-C Dist C65      -C74      .       1.43 Ang.
PLAT432_ALERT_2_G Short Inter X...Y Contact  P3       ..F16              2.42 Ang.
                                          1-x,y,1/2-z  =      2_655 Check
PLAT432_ALERT_2_G Short Inter X...Y Contact  F13      ..C37              2.94 Ang.
                                      3/2-x,3/2-y,1-z  =      7_666 Check
PLAT605_ALERT_4_G Largest Solvent Accessible VOID in the Structure        316 A**3
PLAT860_ALERT_3_G Number of Least-Squares Restraints .............        190 Note
PLAT883_ALERT_1_G No Info/Value for _atom_sites_solution_primary .     Please Do !
```

```
PLAT933_ALERT_2_G Number of OMIT Records in Embedded .res File ...         16 Note
PLAT941_ALERT_3_G Average HKL Measurement Multiplicity ...........        4.5 Low
```

```
   0 ALERT level A = Most likely a serious problem - resolve or explain
   0 ALERT level B = A potentially serious problem, consider carefully
  13 ALERT level C = Check. Ensure it is not caused by an omission or oversight
  39 ALERT level G = General information/check it is not something unexpected

   1 ALERT type 1 CIF construction/syntax error, inconsistent or missing data
  16 ALERT type 2 Indicator that the structure model may be wrong or deficient
   3 ALERT type 3 Indicator that the structure quality may be low
  32 ALERT type 4 Improvement, methodology, query or suggestion
   0 ALERT type 5 Informative message, check
```

It is advisable to attempt to resolve as many as possible of the alerts in all categories. Often the minor alerts point to easily fixed oversights, errors and omissions in your CIF or refinement strategy, so attention to these fine details can be worthwhile. In order to resolve some of the more serious problems it may be necessary to carry out additional measurements or structure refinements. However, the purpose of your study may justify the reported deviations and the more serious of these should normally be commented upon in the discussion or experimental section of a paper or in the "special_details" fields of the CIF. checkCIF was carefully designed to identify outliers and unusual parameters, but every test has its limitations and alerts that are not important in a particular case may appear. Conversely, the absence of alerts does not guarantee there are no aspects of the results needing attention. It is up to the individual to critically assess their own results and, if necessary, seek expert advice.

Publication of your CIF in IUCr journals

A basic structural check has been run on your CIF. These basic checks will be run on all CIFs submitted for publication in IUCr journals (*Acta Crystallographica*, *Journal of Applied Crystallography*, *Journal of Synchrotron Radiation*); however, if you intend to submit to *Acta Crystallographica Section C* or *E* or *IUCrData*, you should make sure that full publication checks are run on the final version of your CIF prior to submission.

Publication of your CIF in other journals

Please refer to the *Notes for Authors* of the relevant journal for any special instructions relating to CIF submission.

PLATON version of 10/08/2020; check.def file version of 06/08/2020

Datablock 2 - ellipsoid plot

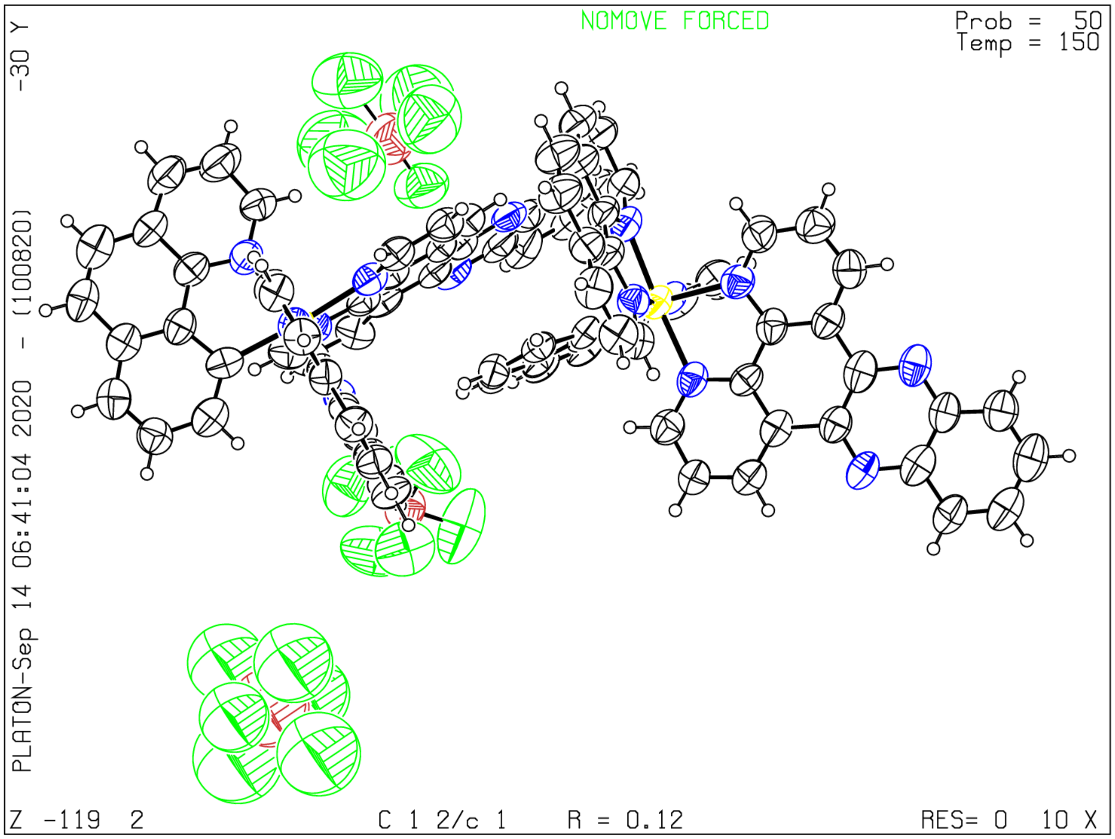

Supplement: Supplementary file 2 — Supporting Information [file ADMA-34-2100245-s002.zip › checkcif-RuZ.pdf]
